# Four-Component Relativistic Calculations of NMR Shielding Constants of the Transition Metal Complexes—Part 2: Nitrogen-Coordinated Complexes of Cobalt

**DOI:** 10.3390/ijms232113178

**Published:** 2022-10-29

**Authors:** Dmitry O. Samultsev, Valentin A. Semenov, Irina L. Rusakova, Leonid B. Krivdin

**Affiliations:** A. E. Favorsky Irkutsk Institute of Chemistry, Siberian Branch of the Russian Academy of Sciences, Favorsky St. 1, Irkutsk 664033, Russia

**Keywords:** four-component relativistic NMR calculations, transition metal complexes, nitrogen-coordinated complexes of cobalt

## Abstract

Both four-component relativistic and nonrelativistic computations within the GIAO-DFT(PBE0) formalism have been carried out for ^15^N and ^59^Co NMR shielding constants and chemical shifts of a number of the nitrogen-coordinated complexes of cobalt. It was found that the total values of the calculated nitrogen chemical shifts of considered cobalt complexes span over a range of more than 580 ppm, varying from −452 to +136 ppm. At that, the relativistic corrections to nitrogen shielding constants and chemical shifts were demonstrated to be substantial, changing accordingly from ca. −19 to +74 ppm and from −68 to +25 ppm. Solvent effects on ^15^N shielding constants and chemical shifts were shown to have contributions no less important than the relativistic effects, namely from −35 to +63 ppm and from −74 to +23 ppm, respectively. Cobalt shielding constants and chemical shifts were found to vary in the ranges of, accordingly, −20,157 to −11,373 ppm and from +3781 to +13,811. The relativistic effects are of major importance in the cobalt shielding constants, resulting in about 4% for the shielding-type contributions, while solvent corrections to cobalt shielding constants appeared to be of less significance, providing corrections of about 1.4% to the gas phase values.

## 1. Introduction

Noble metal complexes have become of primary importance in many areas of modern chemistry; however, the high cost of noble metals essentially hinders their large-scale application and industrialization. In this respect, extensive attention has been paid to the non-noble metal complexes, such as those of cobalt, rhodium, ruthenium, and iridium, providing the most interest and perspective. In particular, cobalt complexes are extensively studied nowadays as potentially important synthetic products capable of exhibiting antiulcer and anti-microbial activities [1,2,3,4,5,6]. The present study deals with cobalt ionic complexes with nitrogen-donor ligands that represent a potential interest in biochemistry.

Nitrogen and cobalt NMR spectroscopy makes up an efficient tool for the structural elucidation of cobalt ammine complexes. Chemical shifts of both NMR-active nitrogen isotopes, ^14^N (*S* = 1) and ^15^N (*S* = 1/2), span over the range of about 900 ppm [7]. The ^59^Co isotope has 100% natural abundance and its chemical shift range is extremely wide, being about 20,000 ppm [8], which is the largest among the known NMR scales. In this regard, accurate theoretical predictions of the NMR chemical shifts provide a powerful tool for the structural elucidation of organic and bioorganic molecules, transition metal complexes, and related species [9,10,11,12,13]. In this respect, NMR chemical shifts, nitrogen and cobalt in particular, represent an undoubted challenge [14,15,16,17,18]. Moreover, cobalt complexes are computationally demanding on their own, as they possess intricate electronic structures characterized, in particular, by the energetically low-lying transitions [19], which assume an important role in electron correlation effects.

This work represents an important part of our study on the computational aspects of the NMR shielding constants of the transition metal complexes. The primary concern of the present study was to estimate the ^15^N NMR chemical shifts (CSs) in a large variety of nitrogen-coordinated cobalt complexes within the framework of density functional theory (DFT) [20]. To the best of our knowledge, despite a good deal of experimental information on the nitrogen chemical shifts of cobalt ammine complexes reported so far [21,22,23,24], the corresponding computational results are practically absent.

In our previous paper [25], the first attempt to estimate the ^15^N NMR chemical shifts of three pentaamine aqua complexes of cobalt(III), rhodium(III), and iridium(III) at the nonrelativistic and four-component relativistic DFT-PBE0 [26,27] levels of theory has been made. The relativistic results appeared to be very close to the experiment. That is why we adopted many aspects of the presented computational protocol in the present study. Moreover, in that work, we found noticeable relativistic corrections to the nitrogen shielding constants (of up to 20 ppm) in the cobalt pentaamine complex as a result of the manifestation of the so-called relativistic “Heavy Atom on Light Atom” (HALA) effect, originally described by Nomura and Takeuchi [28]. In the present paper, we consider relativistic corrections to nitrogen chemical shifts in a large number of nitrogen-coordinated cobalt complexes with different ligands and investigate the role of the HALA effect in more detail. Another part of the present study deals with the calculation of cobalt shielding constants (SCs) in the nitrogen-coordinated cobalt complexes within the same DFT-based methodology. In our previous paper [25], we evaluated the cobalt shielding constant in the [Co(NH_3_)_5_H_2_O]^3+^ complex at the four-component DFT-PBE0 level to be approximately −18,236 ppm, while relativistic correction was found to be 892 ppm, which is 4.8% of the total relativistic value.

Since the mid-1990s, transition metal chemical shifts [29,30] have routinely been calculated at the DFT level, including ^59^Co NMR chemical shifts as the prime goal of such computations [19,31,32,33,34,35,36,37]. The first attempts to calculate ^59^Co NMR chemical shifts using the density functional approach were made by Chan et al. [19]. They calculated ^59^Co chemical shifts and chemical shift anisotropies of several hexacoordinated Co(III) complexes using the sum-over-states density functional perturbation theoretical method using individual gauges localized orbitally (SOS-DFPT-IGLO) [38,39]. From the solid-state NMR data presented in their paper, it followed that they underestimated the isotropic chemical shifts of ^59^Co approximately by two times as compared to the experiment. Later, Chan et al. [31,32] continued the study of the computational protocol for the ^59^Co chemical shifts in hexacoordinated cobalt(III) complexes and, as a result, it was found that hybrid DFT exchange-correlation (XC) functionals were more suitable for the ^59^Co shielding calculations, as compared to the pure XC functionals. It was also found that the gauge-including atomic orbitals (GIAO) [40,41,42] scheme provided cobalt CSs of a better agreement with the experimental values than the IGLO [38,39] scheme did.

Godbout et al. [33] performed the density functional calculations of the isotropic ^59^Co NMR CSs of some anionic, cationic, and neutral Co(III) complexes using a hybrid XC B3LYP functional [43,44] within the GIAO formalism. Those results appeared to be of average quality, deviating from the experimental data by no more than 2000 ppm for the cobalt shift scale, covering the range of 20,000 ppm. Overall, Godbout et al., reached several important conclusions, namely that (i) *f*-type functions do not appear to be essential for the correct description of a cobalt atom in the calculations of its shielding constant at the DFT level; (ii) the relativistic effects are not important in this case, so there is no need to apply the relativistic level of theory; (iii) there are no systematic differences between shielding constants calculated for anionic and cationic complexes, so the charge field effects are small.

The hybrid density functional theoretical study of ^59^Co NMR chemical shifts and shift tensor components in the hexacoordinated Co(III) porphyrin system has been carried out by Xu and Au-Yeung [34]. They applied the B3LYP/6-311G ** level of theory using experimental geometry and obtained an excellent agreement with the solid-state NMR experimental data. It was found that the diamagnetic shielding of ^59^Co is close to that of the free atom value of 2166 ppm calculated by Malli [45] and that agreement between the HF method and experimental results is very poor, confirming the fact that electron correlation effects play an essential role in cobalt shielding calculations.

Advanced computations of cobalt chemical shifts that covered the range of about 20,000 ppm have been carried out by Grigoleit et al. [35] for the representative series of electron-rich organometallic and high-valent inorganic Co(III) complexes. In that study, the GIAO-DFT-B3LYP approach had been used in both static calculations on the equilibrium geometries as well as in combination with the methods that include zero-point and classical thermal effects. Mean absolute deviations between averaged and experimental *δ*(^59^Co) values reported in Ref. [35] appeared to be of the order of 500–760 ppm over a chemical shift range of almost 20,000 ppm. The authors had come to a very important conclusion about the vibrational and solvent effects on ^59^Co NMR chemical shifts, namely that taking into account the zero-point and thermal averaging effects results in the insignificant deshielding of ^59^Co nuclei. At that, the largest errors originate in the solvation effects, which are to be addressed by means of employing the highest feasible level of theory by implying an appropriate solvation model.

Density functional calculations of ^59^Co NMR chemical shifts using the zeroth-order regular approximation (ZORA) [46] were reported in [36,37]. Thus, Ooms and co-authors [36] presented an experimental solid-state ^13^C and ^59^Co NMR study of five octahedral Co(III) cations, corroborated by ZORA-DFT calculations, which were carried out without taking into account solvent or vibrational corrections. Indeed, calculated CSs agreed well with the experimental values; however, a significant deviation of more than 2300 ppm for [Co(CH_3_)(en)_2_(N_3_)]^+^ (en = ethylenediamine) was found. In another paper by Senn et al. [37], the ligand-field density functional theory (LF-DFT) approach was employed for the calculations of ^59^Co NMR shielding tensor of all four diastereoisomers of *tris*(1,2-ethanediamine) cobalt(III) complex ion, [Co(en)_3_]^3+^. Those results were compared with the conventional ZORA-DFT calculations and with corresponding experimental values. It was found that the LF-DFT approach slightly overestimated (by several hundreds of ppm) while ZORA-DFT slightly underestimated the experimental values.

In this paper, we shall employ a full four-component GIAO-DFT approach with hybrid functional PBE0 to calculate ^59^Co NMR chemical shifts in a wide series of nitrogen-coordinated cobalt complexes and to pinpoint the typical magnitudes of relativistic effects for cobalt shielding constants and chemical shifts. Predicted cobalt chemical shifts will allow one to determine a range of informative ^59^Co NMR spectra for the related nitrogen-coordinated cobalt complexes.

## 2. Results and Discussion

**Figure 1:**
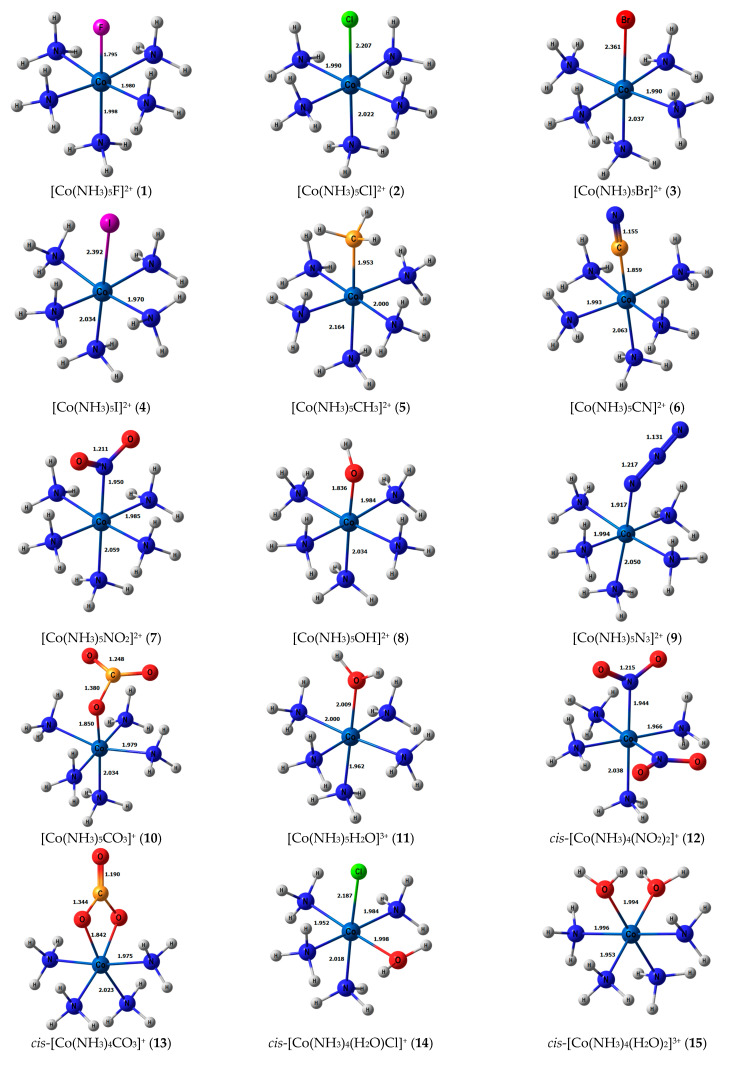

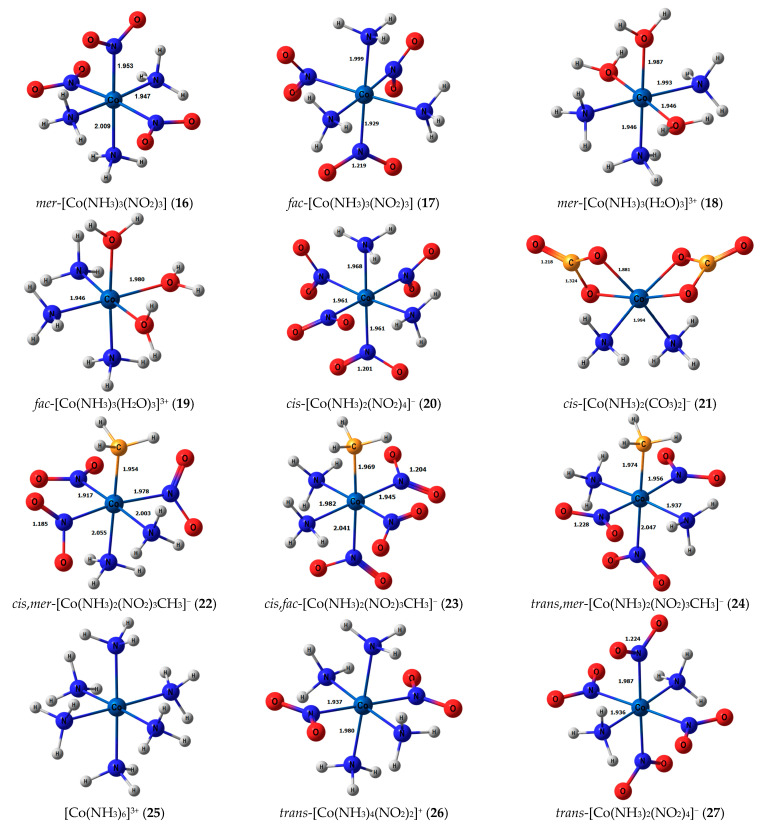
Equilibrium geometries of the nitrogen-coordinated cobalt complexes **1**–**27**, obtained at the PBE0/ATZP level of theory.

Stereochemical structures, together with calculated geometric parameters of complexes **1**–**27** optimized at the DFT-PBE0/ATZP level, are shown in Figure 1. The corresponding calculated ^15^N shielding constants (*σ*_tot_), together with theoretical (*δ*_tot_) and experimental chemical shifts (*δ*_exp_), are given in Table 1.

In Table 1, *σ*_GP_, Δ_solv_, Δ_rel_, and *σ*_tot_ stand, respectively, for the gas phase values of nitrogen shielding constants, solvent corrections to nitrogen SCs, relativistic corrections to nitrogen SCs, and total values of nitrogen SCs, while *δ*_tot_ and *δ*_exp_ are the total theoretical and experimental nitrogen chemical shifts. Basic gas phase values were calculated at the DFT-PBE0 level of theory using relativistic Dyall’s core-valence basis set of triple zeta quality, dyall.cv3z [47,48], on cobalt atoms, aug-pcS-2 [49] on nitrogen atoms, aug-cc-pVDZ [50,51] on oxygen atoms, pc-2 [52,53] on carbon atoms, and pc-1 [52,53] on hydrogens. For the sake of convenience, the mentioned basis set scheme will be referred to as BaS. In all calculations of shielding constants, we have used GIAO formalism to treat the gauge origin problem [13,54].

Used in our calculations, the hybrid PBE0 functional represents the combination of the PBE generalized gradient functional [55], in which all parameters (except those related to the local spin density) are fundamental constants, with a 25% admixture of the Hartree−Fock (HF) exchange.

Solvent corrections to the ^15^N SCs were estimated as the differences between the SC values obtained at the DFT-PBE0/BaS level within the polarizable continuum model using the integral equation formalism (IEF-PCM) [56,57], specified for the H_2_O solvent, and the GP values, evaluated at the same level of theory.

Relativistic corrections to the ^15^N SCs were evaluated within the GIAO-DFT-PBE0 method as the differences between the four-component relativistic values and the approximated nonrelativistic values. In all the four-component calculations, we generated the small-component basis space by applying the unrestricted kinetic balance (UKB) [58] to the large-component basis set. This was done in order to approximate the magnetic kinetic balance (MKB) condition [59], because, as was found by Olejniczak et al. [60], the GIAOs make MKB an atomic one [61]. As a result, it becomes possible to obtain the magnetic balance by extending the orbitals, retrieved from a self-consistent field (SCF) calculation with the restricted kinetic balance (RKB) condition [58] by extending with their UKB complement. In both relativistic and approximated nonrelativistic four-component calculations, we have used the same basis set scheme, BaS, as in the nonrelativistic calculations. The only difference is that all basis sets were taken in an uncontracted form, BaS(un). This is due to the poor suitability of the nonrelativistic contraction schemes for the four-component relativistic calculations [62].

**Figure 2:**
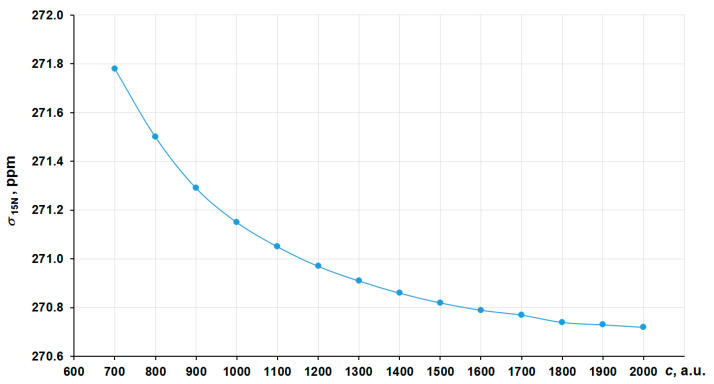
Convergence of the ^15^N NMR shielding constant of [Co(NH_3_)_6_]^3+^ to the *c*^∞^ limit in the GIAO-4DFT-PBE0/BaS(uc) calculation.

In order to obtain the correct nonrelativistic limit, we have investigated the convergence of the nitrogen shielding constant in [Co(NH_3_)_6_]^3+^ with the increasing of the speed of light value (*c* ~ 137.036 a.u.) by several times, namely starting from 700 a.u. (~5*c*) to 2000 a.u. (~14*c*) in the four-component GIAO-DFT-PBE0 calculations. The corresponding graph is presented in Figure 2. In that way, the values at 1800–2000 a.u. can solidly be regarded as the converged ones. Thus, we have chosen *c* = 1800 a.u. (we will call it the “13c scheme”) to calculate the approximated nonrelativistic values of nitrogen shielding constants.

The total values of nitrogen SCs in Table 1 represent the sum of *σ*_GP_, Δ_solv_, and Δ_rel_:

*σ*_tot_ = *σ*_GP +_ Δ_solv +_ Δ_rel_ (1)

The total values of nitrogen chemical shifts *δ*_tot_ in Table 1 were calculated using the linear regression analysis [63,64,65]. The strategy of this approach consists of the mapping of the observed chemical shifts onto the predicted shielding constants, in which the relationship is simulated by the linear model:

*σ* = *Aδ* + *B* (2)

**Figure 3:**
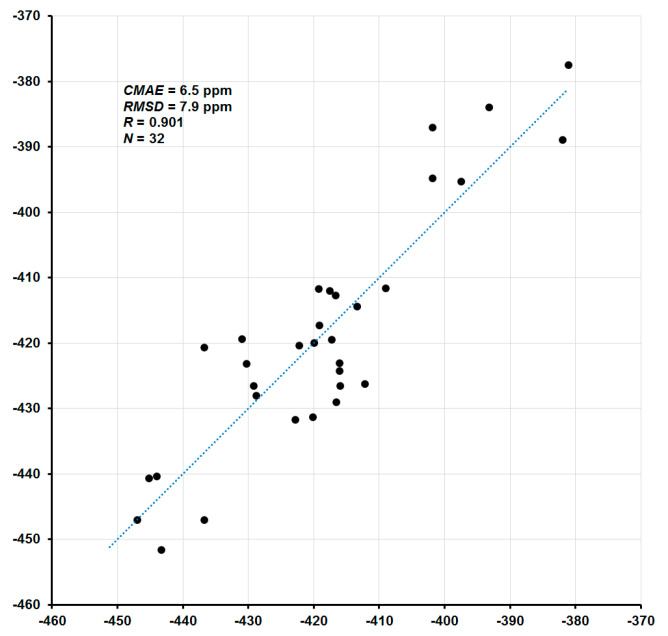
Correlation plot of calculated ^15^N NMR chemical shifts (ppm) versus experiment.

The coefficient *A* represents a slope (the tangent of the line angle) and *B* is the intercept of the model with the *σ*-axis (which corresponds to the approximated shielding constant of the reference compound). In the absence of systematic errors, coefficient *A* takes the value of −1, while *B* becomes *σ*_ref_. In the present case, total nitrogen SCs (*σ*_tot_) were mapped to available experimental CSs (*δ*_exp_), and the parameters of the linear regression model were obtained as *A* = −1.1045 and *B* = −179.90. The correlation plot of the final calculated ^15^N NMR chemical shifts (*δ*_tot_) versus corresponding experimental values (*δ*_exp_) is shown in Figure 3. In general, given that total CSs span the range from −451.6 to +136.1 ppm (a range of more than 580 ppm), the agreement of the calculated nitrogen CSs with the experiment is rather good: the correlation coefficient is 0.901, the corrected mean absolute error (CMAE) is 6.5 ppm, and the mean absolute percentage error (MAPE) is only 1.14%.

**Figure 4:**
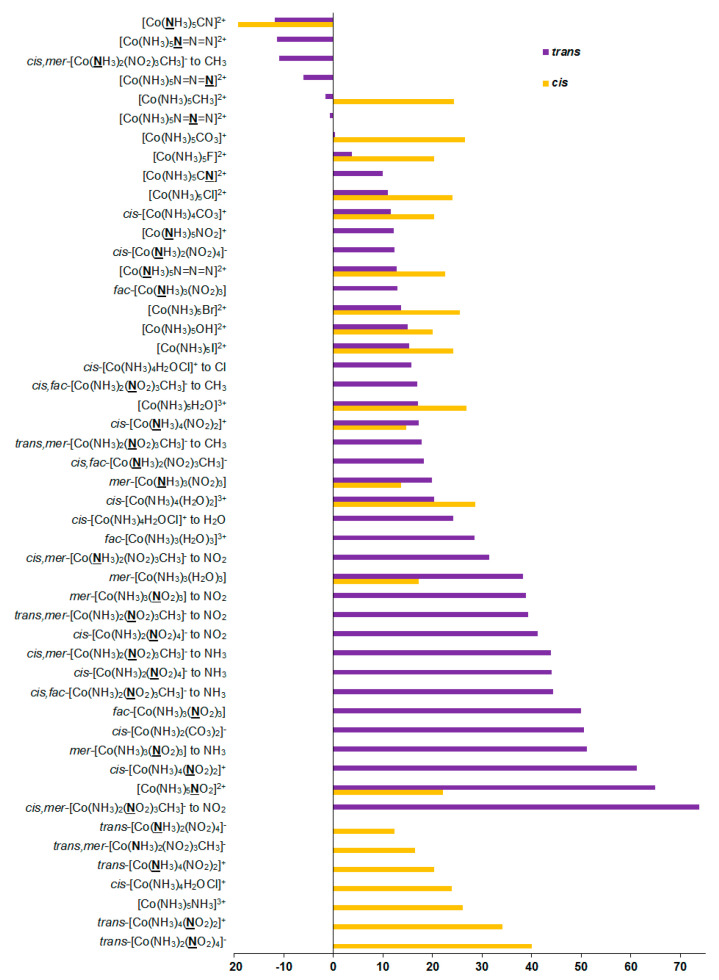
Relativistic corrections to the ^15^N shielding constants, ppm.

As can be seen in Table 1, the relativistic corrections to nitrogen shielding constants can be substantial, varying from −19 ppm in [Co(**N**H_3_)_5_CN]^2+^ (*cis*-orientation) to 74 ppm in *cis*,*mer*-[Co(NH_3_)_2_(**N**O_2_)_3_CH_3_]^−^ (*trans*-orientation to NO_2_), see Figure 4. In an absolute value, the relativistic corrections to the nitrogen SCs can reach up to 37% of the relativistic SCs. Apparently, large relativistic corrections to nitrogen shielding constants are due to the well-known SO-HALA effect [66] on nitrogen shieldings initiated by the neighboring cobalt atom.

The mechanism of the SO-HALA effect consists of the interaction of the spin-orbit coupling (SOC) at the heavy atom with the magnetic dipole at the light nucleus. Namely, in the presence of an external magnetic field, SOC produces additional electronic spin polarization, which propagates to the light nucleus and changes the magnetic field at the light nucleus via the FC interaction [67,68]. Due to its nonlocal character, the sign and magnitude of the SO-HALA effect can give information about the electronic structure of the heavy atom and its surroundings [69,70,71,72]. In particular, it reflects the coordination environment of the heavy atom center [73] and gives information on the polar/covalent character of the heavy–light atom bond [72,74]. Moreover, from the sign of the SO-HALA effect, one can deduce the character of the frontier orbitals participating in the SO-HALA mechanism. Namely, the deshielding SO-HALA effect is associated with the occupied *σ*-type heavy–light atom bonding molecular orbitals, while the *π*-type orbitals provide a shielding-type SO-HALA effect [68,75]. These findings were confirmed for hydrogen and carbon shielding constants by Kaupp et al. [76,77,78,79], Bagno et al. [80], and Ruiz-Morales et al. [81], who carried out the full four-component DFT investigations of the SO-HALA effects in the transition-metal complexes involving molecular orbital analysis.

Based on our results, it follows that the magnitude and sign of this effect are dependent on the electronic nature of ligands. Apparently, this can be explained by the concept discussed above. Indeed, the sign of the SO-HALA effect observed in the series of cobalt complexes **1**–**27** is likely defined by the interplay of the involvement of the occupied *σ*- and *π*-type metal *d*-orbitals in the bonding metal orbital with the NMR spectator nitrogen atom, which should be influenced by the electronic nature of the ligands sharing the same cobalt–nitrogen bonding orbital. The positive sign of most of the relativistic corrections to nitrogen SCs in compounds **1**–**27** is due to the fact that the negative paramagnetic contributions decrease in their absolute values when going to the relativistic consideration, while diamagnetic terms stay practically unchanged. This indicates that the observed SO-HALA effect provides shielding-type contributions to the paramagnetic terms, resulting in their increase if one takes into account their negative sign. Based on these observations, the occupied *π*-type metal *d*-orbitals play a predominant role in the observed SO-HALA effect on the nitrogen SCs in most of the considered cobalt complexes **1**–**27**. Otherwise, the SO-HALA effect is probably governed by the *σ*-type occupied metal *d*-orbitals.

Based on the simplified IUPAC formula for chemical shift [82,83], we have also estimated the relativistic corrections to nitrogen chemical shifts as the differences between the relativistic correction to nitrogen shielding constant in nitrogen reference compound (nitromethane, CH_3_NO_2_) and those in cobalt complexes **1**–**27**:

Δ_rel_*δ* = Δ_rel_*σ*_ref_ − Δ_rel_*σ* (3)

According to the present results, relativistic correction to the nitrogen SC in CH_3_NO_2_ is only 5.3 ppm. Subtracting the relativistic corrections to nitrogen SCs in compounds **1**–**27** from this value (5.3 ppm) gives relativistic corrections to the nitrogen CSs of ca. −68 to +25 ppm.

An important conclusion that can be arrived at from these figures is that the relativistic SO-HALA corrections to the nitrogen chemical shifts in the vast majority of cases are negative (i.e., of the shielding type), shifting nitrogen signals to a higher field. The magnitude of the SO-HALA effect on nitrogen is dependent on the nature of ligands and sometimes it can be rather substantial, so we recommend applying the relativistic level of theory when calculating nitrogen CSs in the nitrogen-coordinated complexes of cobalt.

**Figure 5:**
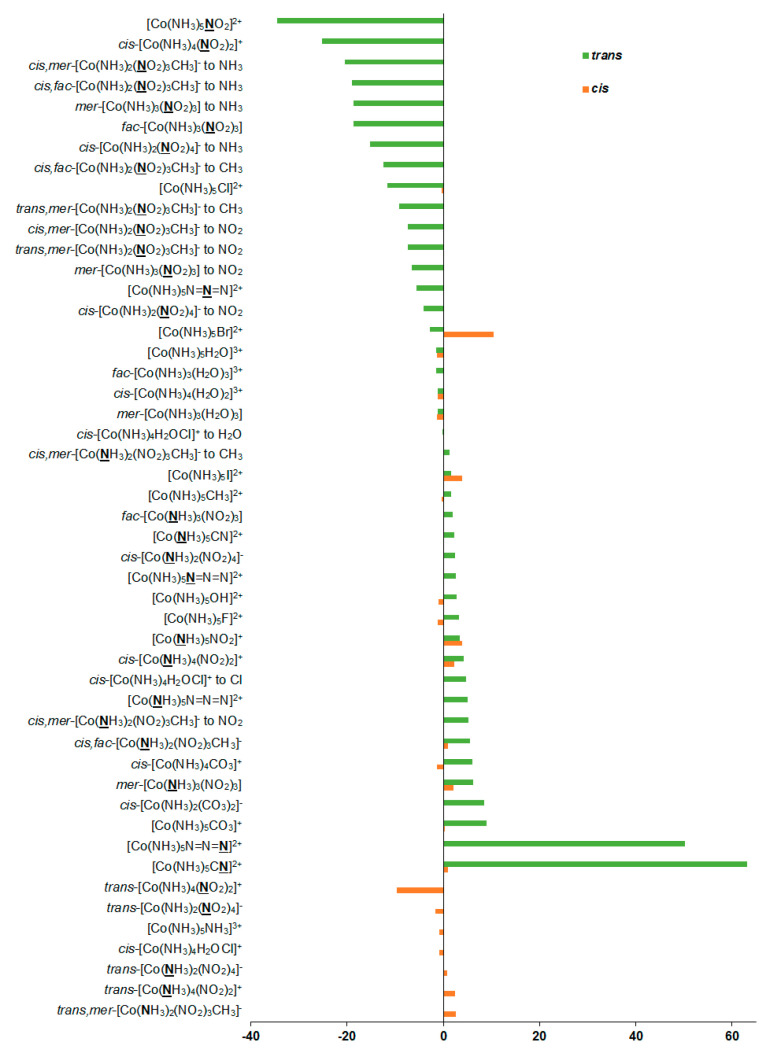
Solvent corrections to the ^15^N NMR shielding constants, ppm.

Additionally, we have evaluated solvent corrections to the nitrogen SCs and CSs; see Table 1 and Figure 5. Solvent corrections to SCs were found to be of −34.6 to +63.2 ppm in this series. Based on the solvent correction to nitrogen SC in nitromethane of −11.2 ppm (Δ_solv_*σ*_ref_), we estimated the solvent corrections to nitrogen CSs in the whole series of **1**–**27** as:

Δ_solv_*δ* = Δ_solv_*σ*_ref_ − Δ_solv_*σ* (4)

Solvent corrections to nitrogen CSs were found to be in the range of −74.4 to +23.4 ppm. In view of the significance of solvent corrections to nitrogen CSs, we suggest taking them into account when calculating nitrogen CSs in the nitrogen-coordinated complexes of cobalt.

Additionally, we have calculated ^59^Co NMR shielding constants and chemical shifts within the same computational protocol as was used for nitrogen. However, in view of the lack of experimental data for cobalt NMR, we did not apply the linear regression analysis for cobalt chemical shifts. Instead, we used the approximated IUPAC formula [82,83]:

*δ*_tot_ = *σ*_tot_(ref) − *σ*_tot_, (5)

where *σ*_tot_(ref) is the total value of cobalt SC of the reference [Co(CN)_6_]^3−^, and *σ*_tot_ is the total cobalt SC of the given compound. These results are presented in Table 2.

**Figure 6:**
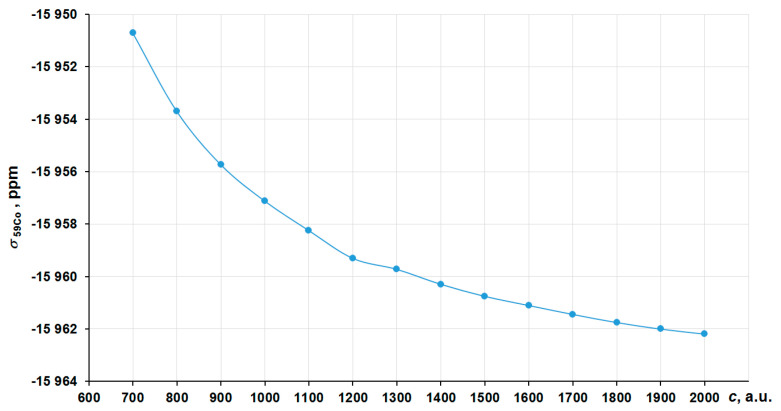
Convergence to the *c*^∞^ limit for the ^59^Co NMR shielding constant in [Co(CN)_6_]^3−^ in the framework of the GIAO-4DFT-PBE0/BaS(uc) calculation.

To calculate the correct nonrelativistic values of *σ* (which are needed for the evaluation of Δ_rel_*σ*), we have studied the convergence of the cobalt *σ* value in the hexacyanocobaltate (III) anion [Co(CN)_6_]^3−^ with the increasing of the speed of light, in the same way as was done for the nitrogen SC. The corresponding graph is presented in Figure 6.

As can be seen in Figure 6, the calculated *σ* varies insignificantly (within 1 ppm) starting from 1800 a.u. To calculate the nonrelativistic limit for the cobalt shielding, we have also chosen the “13c scheme”.

**Figure 7:**
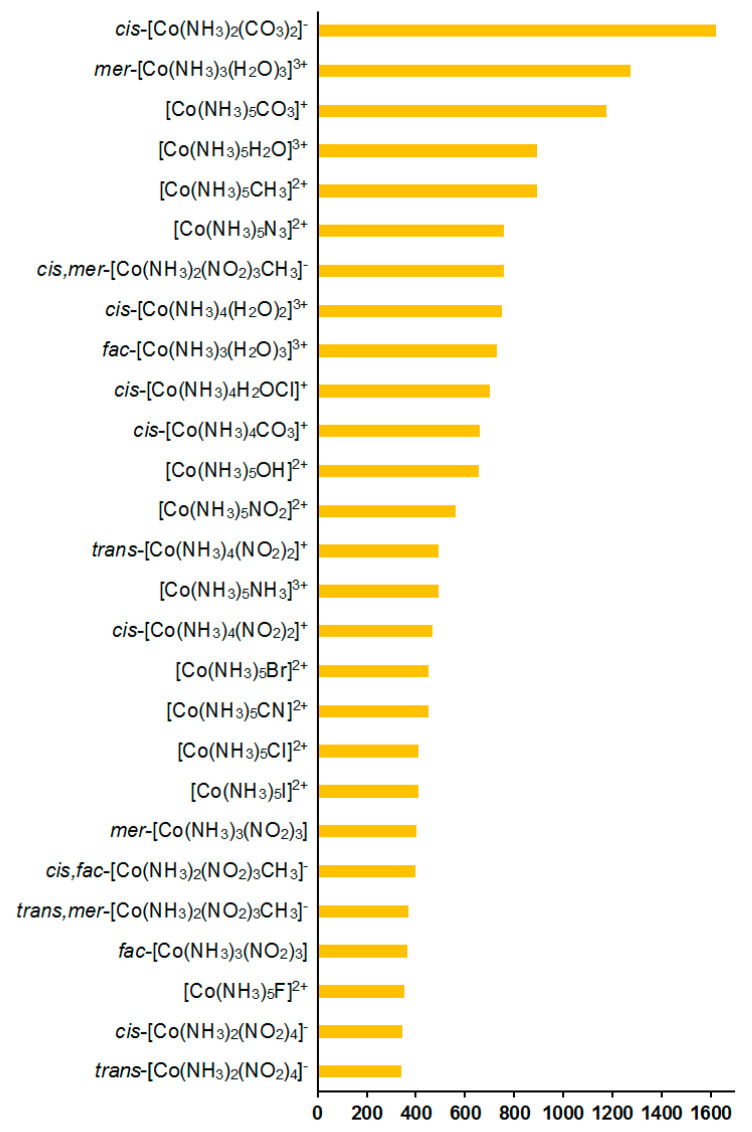
Relativistic corrections to the ^59^Co NMR shielding constants, ppm.

As follows from Table 2, the total calculated SCs of cobalt (*σ*_tot_) are negative, varying from −20,157 to −11,373 ppm, depending on ligands. The relativistic effects were found to play a significant role for ^59^Co SCs, providing, on average, a shielding-type contribution of about 4% in the range of 2–10% of the total values; see Figure 7.

At that, the total cobalt CSs (*δ*_tot_) span over the range from 3781 to 13811 ppm. Given such a wide range of cobalt CSs, it is interesting to compare calculated values with the experimental data whenever it is possible. Having no experimental data for the whole series of considered compounds, we can at least make a comparison for a couple of cobalt complexes; namely, for *cis*-[Co(NH_3_)_4_CO_3_]^+^ (**13**) and [Co(NH_3_)_6_]^3+^ (**25**). According to the solid-state NMR data, the cobalt chemical shifts of compounds **13** and **25** referenced to [Co(CN_6_)]^3−^ are 9691 ppm [33] (9700 ppm [19]) and 8153 ppm [33] (8176 ppm [35]), respectively. Our total calculated values for these compounds are, accordingly, 9194.0 and 9223.1 ppm. Hence, the discrepancy between our theoretical values and the solid-state NMR experimental values for compounds **13** and **25** is about 500 (5% to *δ*_tot_) and 1000 ppm (11% to *δ*_tot_), respectively. Based on the analysis of previous calculations of ^59^Co NMR CSs [19,33,35,36], we have achieved rather good accuracy for the calculated cobalt chemical shifts in this paper.

The relativistic corrections can be estimated as 2–14% with an average relative value of about 5%, which is far from being negligible. In that way, we do not agree with Godbout and Oldfield [33] that taking into account relativistic effects when calculating cobalt chemical shifts is not essential to reproduce the experimental data. On the contrary, the relativistic level of theory is essentially important, because relativistic corrections to ^59^Co NMR chemical shifts of cobalt complexes may be of minor significance in some compounds, but can reach up to 14% of the total value for other complexes. The magnitude of the relativistic effects is determined by the electronic structure of a compound under consideration, and it is difficult to say a priori whether it is appropriate to neglect the relativistic corrections.

**Figure 8:**
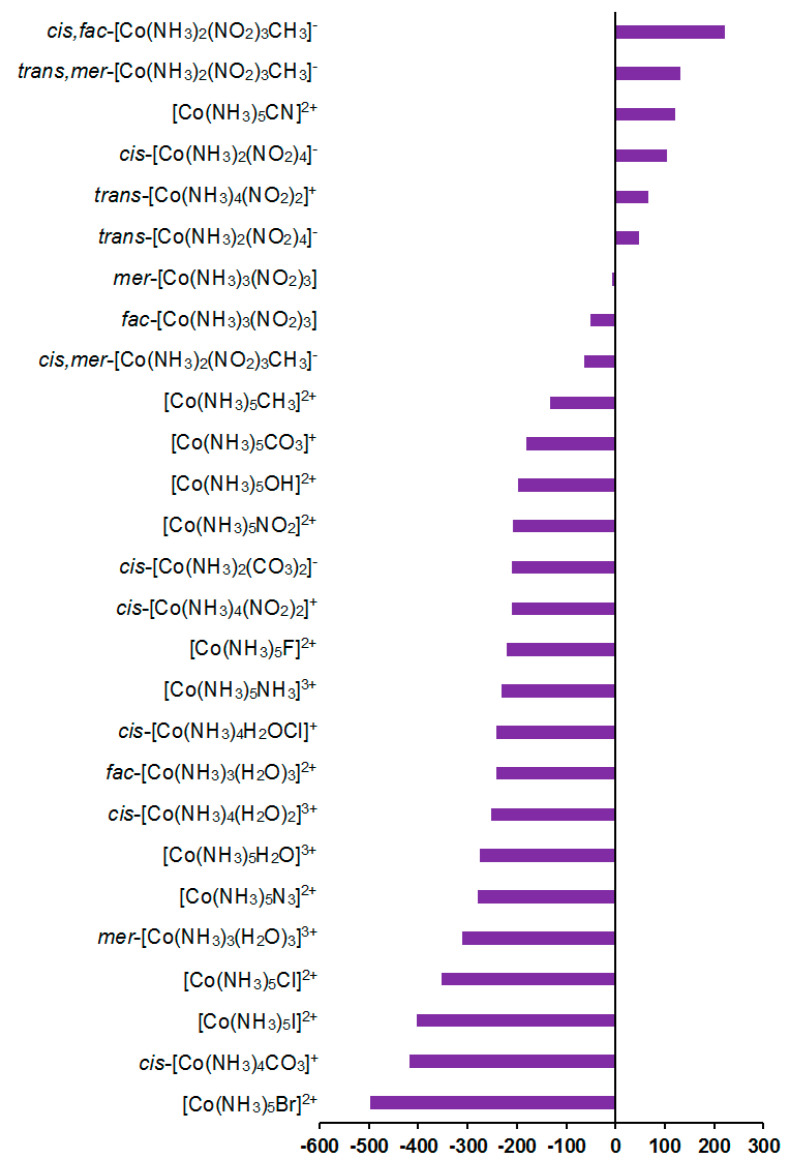
Solvent corrections to the ^59^Co shielding constants, ppm.

Solvent effects in the ^59^Co shielding constants appeared to be less important than the relativistic ones, giving an overall contribution of about 0.1–3.2% to the gas phase values; see Figure 8.

## 3. Materials and Methods

The geometry optimization of the nitrogen-coordinated complexes of cobalt **1**–**27** was performed at the DFT-PBE0/ATZP level using the GAUSSIAN 09 code [84]. As was shown earlier [85], the contribution of relativistic geometry to the resulting ^15^N NMR shielding constants was insignificant and could safely be ignored. Therefore, in the present study, the optimization of the geometric parameters of the studied complexes was carried out at the non-relativistic level of theory. The corresponding Cartesian coordinates of all studied compounds are given in the Supplementary Materials.

All four- and one-component calculations of the ^15^N and ^59^Co NMR shielding constants were performed with the GIAO-DFT-PBE0 method within the Dirac 2016 [86] and Gaussian 09 [84] programs. We have used the DFT formalism at all levels due to the fact that it represented the most suitable tool for our study. Indeed, it takes into account the electron correlation effects via the XC potential and scales as *O*(*n*^4^) [87] at the same time. Despite some disadvantages connected with its inability to systematically improve the accuracy of the results [88] and some issues of triplet instability problems [89], the DFT approach is capable of providing high-quality results, which are comparable to those obtained within the modern ab initio correlated wavefunction-based methods [90].

Therefore, the DFT formalism provides an alternative avenue for the rigorous quantum mechanical calculations of the NMR properties of larger molecular systems, which are beyond the reach and scope of the ab initio correlated wavefunction-based methods. That is why DFT has become very popular in the calculations of the NMR parameters of transition metal complexes (see introduction), and we decided not to digress from a well-proven computational method. Moreover, it is worth noting that, for today, the density functional approach is the only available means of simultaneous taking into account electron correlation and relativistic effects when applied under a relativistic framework.

In all calculations of shielding constants, we used the PBE0 XC functional. This functional indeed provides very accurate results for NMR chemical shifts. In particular, a computational study of the performances of the PBE and PBE0 functionals in application to the shielding constants of light NMR nuclei of the first and second periods was carried out by Adamo and Barone [91]. In that study, they chose quite a large reference set, which included molecules with different hybridizations and chemical environments of the nuclei of interest. It was demonstrated that the PBE0 protocol appeared to be competitive with the low-order perturbation post-HF techniques (such as MP2) for the “well-behaved” systems and provided significantly improved results in the presence of strong correlation effects.

As a result, we carried out geometry optimizations of all nitrogen-coordinated complexes of cobalt using this functional. This was done in line with the recent findings by Giovanetti et al. [92], who studied the effects of the geometry on fluorine spin–spin coupling constants and reached the conclusion that geometry optimization at the same level of theory as that used for the calculation of spin–spin coupling constants generally improves the quality of the final results.

We suppose that this observation can be explained as follows: using the same particular functional in both the geometry optimization and calculation of any triplet property provides the most stable results in the sense of the triplet instability problem [89]. In the present case, we deal with relativistic corrections, which can be expressed as multiple response functions of different orders depending, in particular, on the matrix elements of hyperfine triplet operators of different types [93,94]. Thus, guided by this reasoning, we decided not to introduce any additional factors that can disturb the stability of all relativistic DFT calculations and used the same XC functional in both equilibrium geometry and shielding constant calculations.

## 4. Conclusions

Both four-component relativistic and nonrelativistic computations within the GIAO-DFT(PBE0) formalism were carried out for ^15^N and ^59^Co NMR shielding constants and chemical shifts of a number of the nitrogen-coordinated complexes of cobalt. It was found that the total values of the calculated nitrogen chemical shifts of the cobalt complexes span over the range of more than 580 ppm, varying from −452 to +136 ppm. At that, the relativistic corrections to nitrogen shielding constants and chemical shifts were found to be substantial, varying from −19 to +74 ppm and from −68 to +25 ppm, respectively. The positive sign of the most part of the relativistic corrections to nitrogen SCs was found to be due to the fact that the negative paramagnetic contributions decrease in their absolute values when referring to the relativistic consideration, while diamagnetic terms stay practically unchanged. This indicates that the observed relativistic SO-HALA effect initiated by cobalt gives the shielding-type contributions, resulting in the increasing and decreasing of the nitrogen SCs and CSs, respectively, shifting the latter to a higher field. In this sense, we recommend not neglecting the relativistic level of theory when calculating the nitrogen CSs in the nitrogen-coordinated complexes of cobalt. Solvent corrections to nitrogen SCs and CSs were shown to vary from −34.6 to +63.2 ppm and from −74.4 to +23.4 ppm, accordingly. Cobalt SCs (−20,157 to −11,373 ppm) and CSs (+3781 to +13,811 ppm) were found to be essentially large. The relativistic effects were demonstrated to play a significant role for cobalt SCs, resulting in shielding-type contributions of 4% on average, while solvent corrections to the cobalt SCs appeared to be less significant, affecting the gas phase values by 1.4% on average.

## Figures and Tables

**Table 1:** ^15^N NMR shielding constants and chemical shifts (both in ppm) of amino groups in **1**–**27** calculated at the GIAO-DFT-PBE0 nonrelativistic and four-component relativistic levels of theory.

Cmpd.	Formula	*σ*_GP_	Δ_solv_	Δ_rel_	*σ*_tot_	*δ*_tot_^1^	*δ*_exp_^2^
*trans*							
**1**	[Co(NH_3_)_5_F]^2+^	283.8	3.2	3.8	290.8	−426.2	−451.2
**2**	[Co(NH_3_)_5_Cl]^2+^	271.6	−11.7	11.1	271.0	−408.2	−434.9
**3**	[Co(NH_3_)_5_Br]^2+^	266.2	−2.8	13.7	277.1	−413.8	-
**4**	[Co(NH_3_)_5_I]^2+^	256.5	1.6	15.3	273.4	−410.4	-
**5**	[Co(NH_3_)_5_CH_3_]^2+^	249.5	1.7	−1.6	249.6	−388.9	−382.0
**6**	[Co(NH_3_)_5_CN]^2+^	257.1	2.2	−11.8	247.5	−387.0	−401.8
	[Co(NH_3_)_5_CN]^2+^	−174.8	63.2	10.0	−101.6	−70.9	-
**7**	[Co(NH_3_)_5_NO_2_]^2+^	273.0	3.5	12.2	288.7	−424.3	−416.1
	[Co(NH_3_)_5_NO_2_]^2+^	−239.0	−34.6	65.0	−208.7	26.0	-
**8**	[Co(NH_3_)_5_OH]^2+^	273.5	2.7	15.0	291.2	−426.6	−429.2
**9**	[Co(NH_3_)_5_N_3_]^2+^	269.6	5.1	12.8	287.5	−423.2	−430.3
	[Co(NH_3_)_5_(N=N=N)]^2+^	210.6	2.6	−11.3	201.9	−345.7	-
	[Co(NH_3_)_5_(N=N=N)]^2+^	−17.1	−5.5	−0.7	−23.4	−141.7	-
	[Co(NH_3_)_5_(N=N=N)]^2+^	33.0	50.2	−5.9	77.3	−232.8	-
**10**	[Co(NH_3_)_5_CO_3_]^+^	270.0	9.0	0.4	279.4	−415.9	−437.5
**11**	[Co(NH_3_)_5_H_2_O]^3+^	298.2	−1.5	17.1	313.8	−447.0	−447.0
**12**	*cis*-[Co(NH_3_)_4_(NO_2_)_2_]^+^	269.8	4.2	17.2	291.2	−426.5	−416.0
	*cis*-[Co(NH_3_)_4_(NO_2_)_2_]^+^	−254.0	−25.3	61.2	−218.1	34.6	-
**13**	*cis*-[Co(NH_3_)_4_CO_3_]^+^	267.1	6.0	11.6	284.7	−420.6	−436.7
**14**	*cis*-[Co(NH_3_)_4_(H_2_O)Cl]^+^ to H_2_O	282.9	−0.3	24.2	306.8	−440.7	−445.2
	*cis*-[Co(NH_3_)_4_(H_2_O)Cl]^+^ to Cl	262.7	4.8	15.8	283.3	−419.4	−431.0
**15**	*cis*-[Co(NH_3_)_4_(H_2_O)_2_]^3+^	287.4	−1.2	20.3	306.5	−440.4	−444.0
**16**	*mer*-[Co(NH_3_)_3_(NO_2_)_3_]^3+^	264.7	6.2	20.0	290.9	−426.2	−412.2
	*mer*-[Co(NH_3_)_3_(NO_2_)_3_]^3+^ to NO_2_^−^	−290.2	−6.6	38.9	−257.9	70.6	-
	*mer*-[Co(NH_3_)_3_(NO_2_)_3_]^3+^ to NH_3_	−262.9	−18.7	51.2	−230.4	45.7	-
**17**	*fac*-[Co(NH_3_)_3_(NO_2_)_3_]^3+^	245.8	1.9	12.9	260.6	−398.8	-
	*fac*-[Co(NH_3_)_3_(NO_2_)_3_]^3+^	−282.6	−18.6	49.9	−251.3	64.6	-
**18**	*mer*-[Co(NH_3_)_3_(H_2_O)_3_]^3+^	281.8	−1.2	38.3	318.9	−451.6	−443.3
**19**	*fac*-[Co(NH_3_)_3_(H_2_O)_3_]^3+^	287.4	−1.5	28.5	314.4	−447.5	-
**20**	*cis*-[Co(NH_3_)_2_(NO_2_)_4_]^−^	232.8	2.4	12.4	247.6	−387.1	-
	*cis*-[Co(NH_3_)_2_(NO_2_)_4_]^−^ to NO_2_^−^	−303.8	−4.1	41.3	−266.6	78.5	-
	*cis*-[Co(NH_3_)_2_(NO_2_)_4_]^−^ to NH_3_	−279.7	−15.3	44.0	−251.0	64.4	-
**21**	*cis*-[Co(NH_3_)_2_(CO_3_)_2_]^−^	254.7	8.5	50.6	313.8	−447.0	−436.7
**22**	*cis*,*mer*-[Co(NH_3_)_2_(NO_2_)_3_CH_3_]^−^ to NO_2_^−^	238.4	5.2	31.5	275.1	−411.9	-
	*cis*,*mer*-[Co(NH_3_)_2_(NO_2_)_3_CH_3_]^−^ to CH_3_	226.9	1.2	−10.9	217.2	−359.5	-
	*cis*,*mer*-[Co(NH_3_)_2_(NO_2_)_3_CH_3_]^−^ to NO_2_	−337.2	−7.4	73.8	−270.8	82.3	-
	*cis*,*mer*-[Co(NH_3_)_2_(NO_2_)_3_CH_3_]^−^ to NH_3_	−304.2	−20.4	43.8	−280.8	91.3	-
**23**	*cis*,*fac*-[Co(NH_3_)_2_(NO_2_)_3_CH_3_]^−^	240.5	5.5	18.3	264.3	−402.2	-
	*cis*,*fac*-[Co(NH_3_)_2_(NO_2_)_3_CH_3_]^−^ to CH_3_	−334.8	−12.4	17.0	−330.2	136.1	-
	*cis*,*fac*-[Co(NH_3_)_2_(NO_2_)_3_CH_3_]^−^ to NH_3_	−305.9	−19.0	44.4	−280.5	91.1	-
**24**	*trans*,*mer*-[Co(NH_3_)_2_(NO_2_)_3_CH_3_]^−^ to NO_2_^−^	−322.0	−7.3	39.3	−290.0	99.7	-
	*trans,mer*-[Co(NH_3_)_2_(NO_2_)_3_CH_3_]^−^ to CH_3_	−318.8	−9.1	17.8	−310.1	117.9	-
*cis*							
**1**	[Co(NH_3_)_5_F]^2+^	256.1	−1.2	20.3	275.2	−412.0	−417.5
**2**	[Co(NH_3_)_5_Cl]^2+^	260.3	−0.4	24.1	284	−420.0	−419.9
**3**	[Co(NH_3_)_5_Br]^2+^	264.0	10.4	25.5	299.9	−434.4	-
**4**	[Co(NH_3_)_5_I]^2+^	258.1	3.9	24.2	286.2	−422.0	-
**5**	[Co(NH_3_)_5_CH_3_]^2+^	268.9	−0.3	24.3	292.9	−428.1	−428.8
**6**	[Co(NH_3_)_5_CN]^2+^	271.0	1.0	−19.2	252.8	−391.8	−425.9
**7**	[Co(NH_3_)_5_NO_2_]^2+^	261.3	3.9	22.2	287.4	−423.1	−416.1
**8**	[Co(NH_3_)_5_OH]^2+^	255.7	−1.0	20.1	274.8	−411.7	−419.2
**9**	[Co(NH_3_)_5_N_3_]^2+^	261.0	0.9	22.5	284.4	−420.4	−422.2
**10**	[Co(NH_3_)_5_CO_3_]^+^	254.1	0.3	26.6	281.0	−417.3	−419.1
**11**	[Co(NH_3_)_5_H_2_O]^3+^	271.0	−1.3	26.8	296.5	−431.3	−420.1
**12**	*cis*-[Co(NH_3_)_4_(NO_2_)_2_]^+^	239.1	2.3	14.7	256.1	−394.7	−401.8
**13**	*cis*-[Co(NH_3_)_4_CO_3_]^+^	256.8	−1.3	20.4	275.9	−412.7	−416.7
**14**	*cis*-[Co(NH_3_)_4_(H_2_O)Cl]^+^	260.4	−0.9	23.9	283.4	−419.5	−417.2
**15**	*cis*-[Co(NH_3_)_4_(H_2_O)_2_]^3+^	266.4	−1.1	28.7	294.0	−429.1	−416.6
**16**	*mer*-[Co(NH_3_)_3_(NO_2_)_3_]^3+^	228.4	2.1	13.7	244.2	−384.0	−393.2
**18**	*mer*-[Co(NH_3_)_3_(H_2_O)_3_]^3+^	261.9	−1.4	17.3	277.8	−414.4	−413.4
**24**	*trans,mer*-[Co(NH_3_)_2_(NO_2_)_3_CH_3_]^−^	237.6	2.6	16.5	256.7	−395.3	−397.5
**25**	[Co(NH_3_)_6_]^3+^	271.7	−0.9	26.1	296.9	−431.7	−422.8
**26**	*trans*-[Co(NH_3_)_4_(NO_2_)_2_]^+^	251.9	2.5	20.3	274.7	−411.6	−409.0
	*trans*-[Co(NH_3_)_4_(NO_2_)_2_]^+^	−279.2	−9.6	34.1	−254.7	67.8	-
**27**	*trans*-[Co(NH_3_)_2_(NO_2_)_4_]^−^	223.9	0.8	12.3	237.0	−377.5	−381.1
	*trans*-[Co(NH_3_)_2_(NO_2_)_4_]^−^	−291.2	−1.6	40.1	−252.7	65.9	-

^1^ Linear regression equation: $\delta =\frac{\sigma -B}{A}$, *B* = −179.90, *A* = −1.1045. The points of [Co(NH_3_)_5_F]^2+^ (*trans*-orientation), [Co(NH_3_)_5_Cl]^2+^ (*trans*-orientation), [Co(NH_3_)_5_CN]^2+^ (*cis*-orientation) and [Co(NH_3_)_5_CO_3_]^+^ (*trans*-orientation) were excluded when evaluating the parameters of the linear regression model. ^2^ Experimental values were taken from Ref. [21].

**Table 2:** ^59^Co NMR shielding constants and chemical shifts (ppm) of **1**–**27**, calculated at the GIAO-DFT-PBE0/BaS nonrelativistic and four-component relativistic levels of theory.

Cmpd.	Formula	*σ*_nr_	*σ*_rel_	Δ_rel_*σ* ^1^	Δ_solv_*σ* ^2^	*σ*_tot_ ^3^	*δ*_nr_ ^4^	*δ*_rel_ ^5^	Δ_rel_*δ* ^6^	Δ_solv_*δ* ^7^	*δ*_tot_ ^8^
**1**	[Co(NH_3_)_5_F]^2+^	−10,394.4	−10,039.7	354.7	−221.1	−15,963.4	3675.1	3550.2	−124.8	230.9	3781.1
**2**	[Co(NH_3_)_5_Cl]^2+^	−14,224.8	−13,813.3	411.5	−351.9	−15,479.7	7505.4	7323.8	−181.6	361.7	7685.5
**3**	[Co(NH_3_)_5_Br]^2+^	−15,554.1	−15,102.6	451.5	−496.5	−15,560.0	8834.8	8613.1	−221.7	506.3	9119.4
**4**	[Co(NH_3_)_5_I]^2+^	−15,124.0	−14,715.5	408.5	−403.4	−13,126.6	8404.7	8226.0	−178.7	413.2	8639.2
**5**	[Co(NH_3_)_5_CH_3_]^2+^	−14,331.6	−13,440.4	891.2	−132.8	−13,538.3	7612.3	6951.0	−661.3	142.6	7093.6
**6**	[Co(NH_3_)_5_CN]^2+^	−12,300.9	−11,850.6	450.3	120.1	−13,199.3	5581.6	5361.1	−220.4	−110.2	5250.9
**7**	[Co(NH_3_)_5_NO_2_]^2+^	−15,244.7	−14,681.2	563.5	−207.4	−14,586.5	8525.4	8191.7	−333.6	217.2	8409.0
**8**	[Co(NH_3_)_5_OH]^2+^	−16,051.5	−15,397.3	654.2	−198.4	−15,265.4	9332.1	8907.8	−424.3	208.2	9116.0
**9**	[Co(NH_3_)_5_N_3_]^2+^	−15,959.8	−15,200.1	759.7	−279.8	−15,103.9	9240.5	8710.7	−529.8	289.6	9000.3
**10**	[Co(NH_3_)_5_CO_3_]^+^	−16,649.2	−15,473.8	1175.4	−180.3	−15,008.2	9929.9	8984.4	−945.5	190.1	9174.5
**11**	[Co(NH_3_)_5_H_2_O]^3+^	−19,128.1	−18,236.5	891.6	−274.8	−18,393.9	12,408.8	11,747.0	−661.8	284.6	12,031.6
**12**	*cis*-[Co(NH_3_)_4_(NO_2_)_2_]^+^	−14,130.2	−13,662.1	468.1	−209.8	−13,621.5	7410.9	7172.7	−238.2	219.7	7392.3
**13**	*cis*-[Co(NH_3_)_4_CO_3_]^+^	−15,914.4	−15,255.3	659.1	−418.3	−15,249.1	9195.0	8765.8	−429.3	428.2	9194.0
**14**	*cis*-[Co(NH_3_)_4_(H_2_O)Cl]^+^	−17,032.5	−16,333.1	699.3	−242.1	−16,271.4	10,313.1	9843.7	−469.5	251.9	10,095.6
**15**	*cis*-[Co(NH_3_)_4_(H_2_O)_2_]^3+^	−18,696.1	−17,945.5	750.6	−252.9	−17,942.9	11,976.8	11,456.0	−520.8	262.7	11,718.7
**16**	*mer*-[Co(NH_3_)_3_(NO_2_)_3_]^3+^	−13,349.6	−12,947.8	401.8	−7.1	−12,806.9	6630.3	6458.4	−171.9	17.0	6475.4
**17**	*fac*-[Co(NH_3_)_3_(NO_2_)_3_]^3+^	−13,740.3	−13,375.0	365.3	−50.4	−13,279.2	7021.0	6885.6	−135.5	60.2	6945.8
**18**	*mer*-[Co(NH_3_)_3_(H_2_O)_3_]^3+^	−21,251.8	−19,978.9	1272.8	−311.7	−20,157.4	14,532.5	13,489.5	−1043.0	321.5	13,810.9
**19**	*fac*-[Co(NH_3_)_3_(H_2_O)_3_]^3+^	−19,478.0	−18,746.8	731.2	−242.4	−18,809.3	12,758.7	12,257.4	−501.3	252.2	12,509.6
**20**	*cis*-[Co(NH_3_)_2_(NO_2_)_4_]^−^	−13,551.3	−13,204.7	346.7	105.0	−13,015.0	6832.0	6715.2	−116.8	−95.2	6620.0
**21**	*cis*-[Co(NH_3_)_2_(CO_3_)_2_]^−^	−18,115.3	−16,493.3	1622.0	−209.6	−16,275.4	11,396.0	10,003.8	−1392.1	219.4	10,223.2
**22**	*cis*,*mer*-[Co(NH_3_)_2_(NO_2_)_3_CH_3_]^−^	−12,948.1	−12,188.7	759.4	−63.5	−12,219.8	6228.8	5699.2	−529.6	73.4	5772.6
**23**	*cis*,*fac*-[Co(NH_3_)_2_(NO_2_)_3_CH_3_]^−^	−12,684.5	−12,287.6	396.9	221.1	−12,057.4	5965.2	5798.1	−167.1	−211.3	5586.8
**24**	*trans*,*mer*-[Co(NH_3_)_2_(NO_2_)_3_CH_3_]^−^	−11,844.1	−11,475.9	368.2	132.2	−11,372.5	5124.8	4986.5	−138.3	−122.3	4864.1
**25**	[Co(NH_3_)_6_]^3+^	−15,961.7	−15,470.5	491.2	−232.2	−15,667.9	9242.4	8981.1	−261.4	242.0	9223.1
**26**	*trans*-[Co(NH_3_)_4_(NO_2_)_2_]^+^	−14,042.9	−13,548.9	494.0	67.4	−13,283.8	7323.5	7059.4	−264.1	−57.6	7001.9
**27**	*trans*-[Co(NH_3_)_2_(NO_2_)_4_]^−^	−13,131.6	−12,790.0	341.6	46.7	−12,664.6	6412.3	6300.5	−111.7	−36.9	6263.6

^1^ Δ_rel_*σ* = *σ*_rel_ − *σ*_nr_; ^2^ Δ_solv_*σ* = *σ*_solv_ − *σ*_GP_; ^3^ *σ*_tot_ = *σ*_GP_ + Δ_rel_*σ* + Δ_solv_*σ*; ^4^ *δ*_nr_ = *σ*_nr_(ref) − *σ*_nr_; ^5^ *δ*_rel_ = *σ*_rel_(ref) − *σ*_rel_; ^6^ Δ_rel_*δ* = *δ*_rel_ − *δ*_nr_ ≡ Δ_rel_*σ*(ref) − Δ_rel_*σ*; ^7^ Δ_solv_*δ* = *δ*_solv_ − *δ*_GP_ ≡ Δ_solv_*σ*(ref) − Δ_solv_*σ*; ^8^ *δ*_tot_ = *δ*_rel_ + Δ_solv_*δ* ≡ *δ*_nr_ + Δ_rel_*δ* + Δ_solv_*δ* = *σ*_tot_(ref) − *σ*_tot_.

## Data Availability

Not applicable.

## Supplementary Materials

The following supporting information can be downloaded at: www.mdpi.com/article/10.3390/ijms232113178/s1.
