# Supplementary material for: Four-Component Relativistic Calculations of NMR Shielding Constants of the Transition Metal Complexes—Part 2: Nitrogen-Coordinated Complexes of Cobalt
Source: Int J Mol Sci. 2022 Oct 29;23(21):13178. doi: 10.3390/ijms232113178 (PMC9658221; doi:10.3390/ijms232113178)
Supplement: Supplementary file 1 [file ijms-23-13178-s001.zip › ijms-1994744-supplementary.pdf]

SUPPORTING INFORMATION

Four-component relativistic calculations of NMR shielding constants of the transition metal complexes. Part 2: Nitrogen-coordinated complexes of cobalt

Dmitry O. Samultsev, Valentin A. Semenov, Irina L. Rusakova, and Leonid B. Krivdin *

A. E. Favorsky Irkutsk Institute of Chemistry, Siberian Branch of the Russian Academy of Sciences, Favorsky St. 1, 664033 Irkutsk, Russia

Correspondence: *krivdin_office@irioch.irk.ru*

TABLE OF CONTENTS

Cartesian coordinates (Angstroms) of 1-27, optimized at the PBE0/ATZP level in the IEF-PCM media (chloroform)........ S3
$[Co(NH_3)_5F]^{2+}$........ S3
$[Co(NH_3)_5Cl]^{2+}$........ S4
$[Co(NH_3)_5Br]^{2+}$........ S5
$[Co(NH_3)_5I]^{2+}$........ S6
$[Co(NH_3)_5CH_3]^{2+}$........ S7
$[Co(NH_3)_5CN]^{2+}$........ S8
$[Co(NH_3)_5NO_2]^{2+}$........ S9
$[Co(NH_3)_5OH]^{2+}$........ S10
$[Co(NH_3)_5N_3]^{2+}$........ S11
$[Co(NH_3)_5CO_3]^{+}$........ S12
$[Co(NH_3)_5H_2O]^{3+}$........ S13
cis-$[Co(NH_3)_4(NO_2)_2]^{+}$........ S14
cis-$[Co(NH_3)_4CO_3]^{+}$........ S15
cis-$[Co(NH_3)_4(H_2O)Cl]^{2+}$........ S16
cis-$[Co(NH_3)_4(H_2O)_2]^{3+}$........ S17
mer-$[Co(NH_3)_3(NO_2)_3]$........ S18
fac-$[Co(NH_3)_3(NO_2)_3]$........ S19
mer-$[Co(NH_3)_3(H_2O)_3]^{3+}$........ S20
fac-$[Co(NH_3)_3(H_2O)_3]^{3+}$........ S21
cis-$[Co(NH_3)_2(NO_2)_4]^{-}$........ S22
cis-$[Co(NH_3)_2(CO_3)_2]^{-}$........ S23
cis,mer-$[Co(NH_3)_2(NO_2)_3CH_3]^{-}$........ S24
cis,fac-$[Co(NO_2)_3(NH_3)_2CH_3]^{-}$........ S25
trans,mer-$[Co(NH_3)_2(NO_2)_3CH_3]^{-}$........ S26
$[Co(NH_3)_6]^{3+}$........ S27
trans-$[Co(NH_3)_4(NO_2)_2]^{+}$........ S28
trans-$[Co(NH_3)_2(NO_2)_4]^{-}$........ S29

Cartesian coordinates (Angstroms) of 1-27, optimized at the PBE0/ATZP level in the IEF-PCM media (chloroform).

Complex **1**:

$[Co(NH_3)_5F]^{2+}$

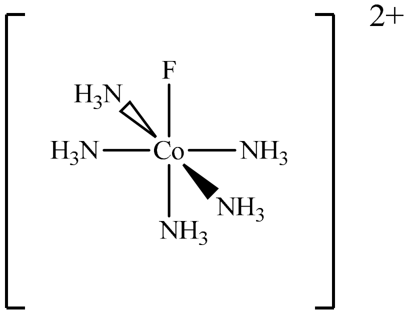

Imaginary frequencies: none
E^0: -1764.5111301 a.u.

Co	0.117326000	0.079822000	0.000000000
N	0.001276000	-0.025062000	1.973850000
N	-1.255707000	1.505083000	0.000000000
N	0.001276000	-0.025062000	-1.973850000
N	1.651326000	1.360652000	0.000000000
N	1.249937000	-1.545470000	0.000000000
H	-0.750248000	-0.705534000	-2.108558000
H	-0.750248000	-0.705534000	2.108558000
H	1.847468000	-1.705387000	0.808938000
H	0.551004000	-2.292128000	0.000000000
H	-1.294175000	2.123578000	-0.807882000
H	-2.125239000	0.966603000	0.000000000
H	-1.294175000	2.123578000	0.807882000
H	1.663388000	1.980056000	-0.808987000
H	1.663388000	1.980056000	0.808987000
H	2.563734000	0.906966000	0.000000000
H	1.847468000	-1.705387000	-0.808938000
H	-0.278841000	0.815802000	2.475173000
H	0.813605000	-0.376361000	-2.477387000
H	-0.278841000	0.815802000	-2.475173000
H	0.813605000	-0.376361000	2.477387000
F	-1.267205000	-1.063176000	0.000000000

Complex **2**:

$[Co(NH_3)_5Cl]^{2+}$

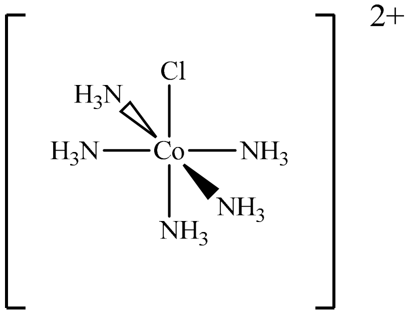

Imaginary frequencies: none
E^0: -2124.7976599 a.u.

Co	0.067527000	0.053224000	0.000000000
N	0.015408000	-0.025164000	1.986387000
N	-1.219656000	1.569132000	0.000000000
N	0.015408000	-0.025164000	-1.986387000
N	1.640361000	1.324181000	0.000000000
N	1.233289000	-1.558961000	0.000000000
H	-0.546241000	-0.847949000	-2.213978000
H	-0.546241000	-0.847949000	2.213978000
H	1.838401000	-1.681222000	0.810265000
H	0.592155000	-2.355336000	0.000000000
H	-1.191436000	2.189295000	-0.807409000
H	-2.146591000	1.137746000	0.000000000
H	-1.191436000	2.189295000	0.807409000
H	1.658626000	1.944298000	-0.808392000
H	1.658626000	1.944298000	0.808392000
H	2.548654000	0.862220000	0.000000000
H	1.838401000	-1.681222000	-0.810265000
H	-0.459391000	0.750475000	2.444883000
H	0.905192000	-0.123587000	-2.472809000
H	-0.459391000	0.750475000	-2.444883000
H	0.905192000	-0.123587000	2.472809000
Cl	-1.667169000	-1.311352000	0.000000000

Complex **3**:

$[Co(NH_3)_5Br]^{2+}$

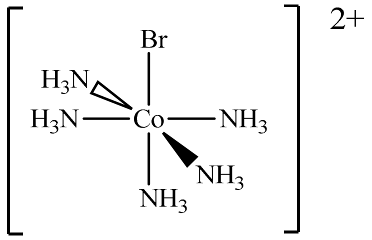

Imaginary frequencies: none
E^0: -4238.5740285 a.u.

Co	0.057879000	0.052031000	0.000000000
N	0.005546000	-0.031036000	1.987265000
N	-1.181295000	1.609909000	0.000000000
N	0.005546000	-0.031036000	-1.987265000
N	1.662771000	1.306776000	0.000000000
N	1.214904000	-1.567839000	0.000000000
H	-0.358223000	-0.950286000	-2.241844000
H	-0.358223000	-0.950286000	2.241844000
H	1.821370000	-1.681154000	0.810715000
H	0.587212000	-2.374712000	0.000000000
H	-1.120720000	2.227833000	-0.807536000
H	-2.130081000	1.229383000	0.000000000
H	-1.120720000	2.227833000	0.807536000
H	1.689960000	1.927889000	-0.807423000
H	1.689960000	1.927889000	0.807423000
H	2.565287000	0.833572000	0.000000000
H	1.821370000	-1.681154000	-0.810715000
H	-0.652640000	0.619153000	2.413392000
H	0.884530000	0.094429000	-2.487821000
H	-0.652640000	0.619153000	-2.413392000
H	0.884530000	0.094429000	2.487821000
Br	-1.826664000	-1.369596000	0.000000000

Complex **4**:

$[Co(NH_3)_5I]^{2+}$

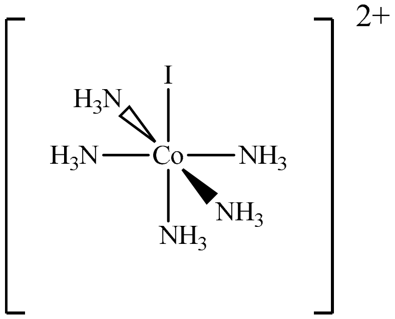

Imaginary frequencies: none
E^0: -8579.2928706 a.u.

Co	0.161215000	0.113751000	-0.000074000
N	0.054220000	0.011358000	1.968425000
N	-1.202081000	1.531957000	-0.000786000
N	0.055992000	0.010660000	-1.968681000
N	1.720168000	1.420779000	0.000032000
N	1.278297000	-1.508886000	0.000871000
H	-0.704002000	-0.672181000	-2.061080000
H	-0.706550000	-0.670834000	2.060233000
H	1.041063000	-2.098737000	-0.794918000
H	2.293086000	-1.405159000	0.000922000
H	-1.827110000	1.417776000	0.795118000
H	-0.889187000	2.502834000	-0.000161000
H	-1.825676000	1.418554000	-0.797920000
H	1.724707000	2.043845000	-0.806104000
H	1.732857000	2.033614000	0.813879000
H	2.637673000	0.978728000	-0.007500000
H	1.041169000	-2.097967000	0.797253000
H	-0.222645000	0.853243000	2.466504000
H	0.871992000	-0.347094000	-2.457831000
H	-0.221415000	0.852017000	-2.467348000
H	0.869480000	-0.347177000	2.458214000
I	-1.686993000	-1.404635000	-0.000637000

Complex **5**:

$[Co(NH_3)_5CH_3]^{2+}$

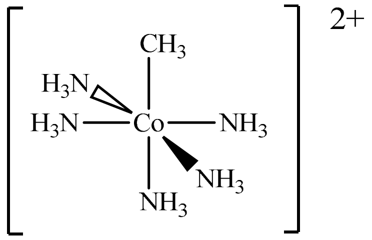

Imaginary frequencies: none
E^0: -1704.5615671 a.u.

Co	-1.239297000	0.757451000	0.000000000
N	-1.212751000	0.679934000	1.993917000
N	-3.215826000	1.035663000	0.000000000
C	-1.581643000	-1.165139000	0.000000000
N	-1.212751000	0.679934000	-1.993917000
N	-0.957896000	2.903353000	0.000000000
H	-0.671350000	-0.122381000	-2.311774000
H	-0.671350000	-0.122381000	2.311774000
H	1.223106000	0.866695000	0.806916000
H	0.974844000	-0.516550000	0.000000000
H	-3.572752000	1.547301000	-0.805148000
H	-3.710043000	0.144302000	0.000000000
H	-3.572752000	1.547301000	0.805148000
H	-1.372273000	3.373787000	-0.802930000
H	-1.372273000	3.373787000	0.802930000
H	0.012757000	3.212386000	0.000000000
H	-2.159981000	-1.448746000	-0.883586000
H	-2.159981000	-1.448746000	0.883586000
H	1.223106000	0.866695000	-0.806916000
H	-2.131242000	0.538271000	2.410105000
H	-0.820028000	1.489847000	-2.471324000
H	-2.131242000	0.538271000	-2.410105000
H	-0.820028000	1.489847000	2.471324000
N	0.740375000	0.474796000	0.000000000
H	-0.662229000	-1.756345000	0.000000000

Complex **6**:

$[Co(NH_3)_5CN]^{2+}$

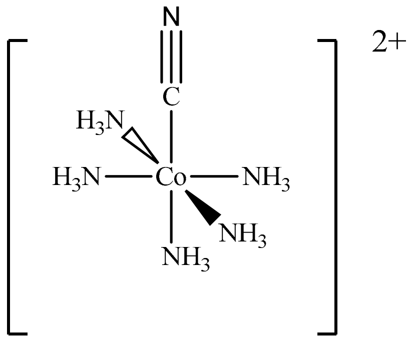

Imaginary frequencies: none
E^0: -1757.4454222 a.u.

Co	0.039238000	0.042261000	0.000000000
N	-0.036431000	-0.049830000	1.989357000
N	-1.190279000	1.611774000	0.000000000
C	-1.454394000	-1.065173000	0.000000000
N	-0.036431000	-0.049830000	-1.989357000
N	1.679804000	1.292313000	0.000000000
H	-0.283775000	-0.998092000	-2.273491000
H	-0.283775000	-0.998092000	2.273491000
H	1.786825000	-1.697953000	0.810176000
H	0.574726000	-2.420787000	0.000000000
H	-1.106150000	2.227648000	-0.807406000
H	-2.156268000	1.279723000	0.000000000
H	-1.106150000	2.227648000	0.807406000
H	1.720801000	1.916052000	-0.805155000
H	1.720801000	1.916052000	0.805155000
H	2.578955000	0.812235000	0.000000000
H	1.786825000	-1.697953000	-0.810176000
H	-0.777917000	0.522143000	2.391126000
H	0.812387000	0.194687000	-2.498051000
H	-0.777917000	0.522143000	-2.391126000
H	0.812387000	0.194687000	2.498051000
N	1.177163000	-1.595872000	0.000000000
N	-2.369982000	-1.769330000	0.000000000

Complex **7**:

$[Co(NH_3)_5NO_2]^{2+}$

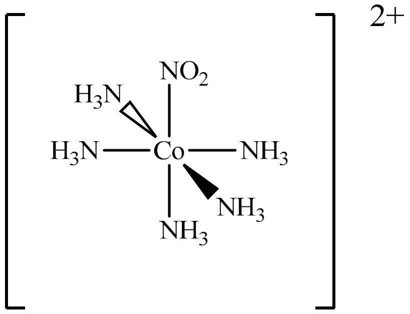

Imaginary frequencies: none
E^0: -1869.6878239 a.u.

Co	0.025519000	0.027628000	0.000000000
N	0.002823000	0.002188000	1.985008000
N	-1.295386000	1.526212000	0.000000000
N	-1.491655000	-1.198007000	0.000000000
N	0.002823000	0.002188000	-1.985008000
N	1.621744000	1.328202000	0.000000000
H	-0.758280000	-0.654381000	-2.208266000
H	-0.758280000	-0.654381000	2.208266000
H	1.817278000	-1.668689000	0.808533000
H	0.652404000	-2.448113000	0.000000000
H	-1.239762000	2.144955000	-0.807353000
H	-2.254528000	1.175904000	0.000000000
H	-1.239762000	2.144955000	0.807353000
H	1.645870000	1.948158000	-0.808020000
H	1.645870000	1.948158000	0.808020000
H	2.531386000	0.868867000	0.000000000
O	-1.935562000	-1.557895000	-1.067262000
O	-1.935562000	-1.557895000	1.067262000
H	1.817278000	-1.668689000	-0.808533000
H	-0.220686000	0.877273000	2.454832000
H	0.842204000	-0.337914000	-2.449913000
H	-0.220686000	0.877273000	-2.454832000
H	0.842204000	-0.337914000	2.449913000
N	1.202614000	-1.587928000	0.000000000

Complex **8**:

$[Co(NH_3)_5OH]^{2+}$

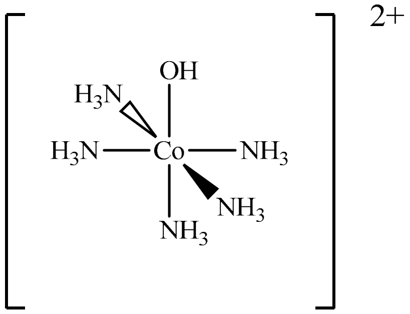

Imaginary frequencies: none
*E*⁰: -1740.4818048 a.u.

Co	0.064279000	0.048346000	-0.029256000
N	-0.060243000	-0.032841000	1.950854000
N	-1.262081000	1.523788000	-0.004832000
O	-1.323264000	-1.149639000	0.079971000
N	0.060045000	-0.053614000	-2.013946000
N	1.641150000	1.332262000	-0.006683000
H	-0.267106000	-0.982047000	-2.280444000
H	-0.823313000	-0.705812000	2.063350000
H	1.877084000	-1.662919000	0.782495000
H	0.504345000	-2.297075000	0.129649000
H	-1.480518000	1.970244000	-0.893508000
H	-2.126288000	1.077787000	0.305930000
H	-1.078163000	2.292537000	0.637568000
H	1.534489000	2.126302000	-0.636190000
H	1.814078000	1.744011000	0.908866000
H	2.526641000	0.902235000	-0.269009000
H	-1.958834000	-1.180985000	-0.641645000
H	1.740476000	-1.800226000	-0.824677000
H	-0.339227000	0.812109000	2.445389000
H	0.963127000	0.070780000	-2.469341000
H	-0.567349000	0.593744000	-2.487865000
H	0.741277000	-0.393394000	2.464419000
N	1.214165000	-1.568912000	0.015645000

Complex **9**:

$[Co(NH_3)_5N_3]^{2+}$

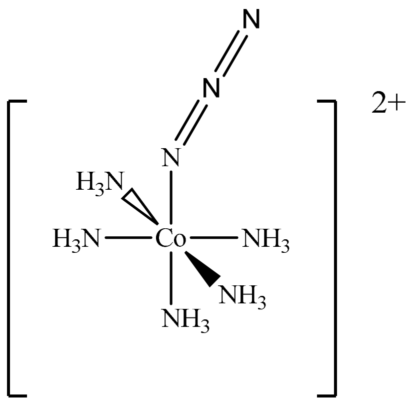

Imaginary frequencies: none
E^0: -1828.7720178 a.u.

Co	-0.028860000	0.084870000	0.000000000
N	-0.147807000	0.061125000	1.988647000
N	-1.005433000	1.823359000	0.000000000
N	-1.806363000	-0.634258000	0.000000000
N	-0.147807000	0.061125000	-1.988647000
N	1.783622000	1.042138000	0.000000000
H	0.258368000	-0.751301000	-2.449636000
H	0.258368000	-0.751301000	2.449636000
H	1.470597000	-1.861473000	0.806240000
H	0.204102000	-2.473768000	0.000000000
H	-0.868891000	2.426862000	-0.808760000
H	-1.991698000	1.554375000	0.000000000
H	-0.868891000	2.426862000	0.808760000
H	1.924634000	1.648403000	-0.806403000
H	1.924634000	1.648403000	0.806403000
H	2.591171000	0.420907000	0.000000000
H	1.470597000	-1.861473000	-0.806240000
H	-1.147958000	0.007099000	2.187625000
H	0.214090000	0.865518000	-2.498281000
H	-1.147958000	0.007099000	-2.187625000
H	0.214090000	0.865518000	2.498281000
N	0.869581000	-1.696233000	0.000000000
N	-1.959682000	-1.841804000	0.000000000
N	-2.141277000	-2.958364000	0.000000000

Complex **10**:

$[Co(NH_3)_5CO_3]^+$

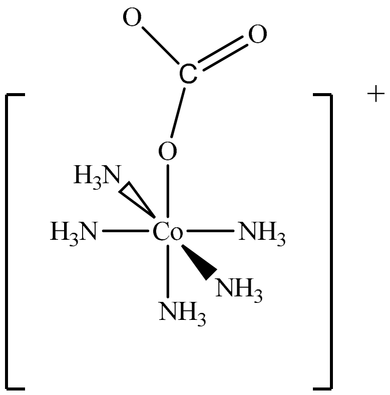

Imaginary frequencies: none
E^0: -1928.6530535 a.u.

Co	-1.015489000	1.062744000	0.000000000
N	-1.148847000	0.772405000	1.952946000
N	-3.020610000	1.177377000	0.000000000
C	-2.084654000	-1.284428000	0.000000000
N	-1.148847000	0.772405000	-1.952946000
N	-0.988231000	3.096478000	0.000000000
H	-0.277010000	0.489770000	-2.389757000
H	-0.277010000	0.489770000	2.389757000
H	1.440072000	1.303539000	0.816294000
H	1.048249000	-0.076807000	0.000000000
H	-3.408102000	1.645196000	-0.814740000
H	-3.422171000	0.229562000	0.000000000
H	-3.408102000	1.645196000	0.814740000
H	-1.444154000	3.504554000	-0.811971000
H	-1.444154000	3.504554000	0.811971000
H	-0.044191000	3.472982000	0.000000000
O	-2.614894000	-1.391326000	-1.125070000
O	-2.614894000	-1.391326000	1.125070000
H	1.440072000	1.303539000	-0.816294000
H	-1.739973000	-0.109921000	1.909104000
H	-1.582408000	1.448928000	-2.574953000
H	-1.739973000	-0.109921000	-1.909104000
H	-1.582408000	1.448928000	2.574953000
N	0.954623000	0.942461000	0.000000000
O	-0.802163000	-0.775272000	0.000000000

Complex **11**:

$[Co(NH_3)_5H_2O]^{3+}$

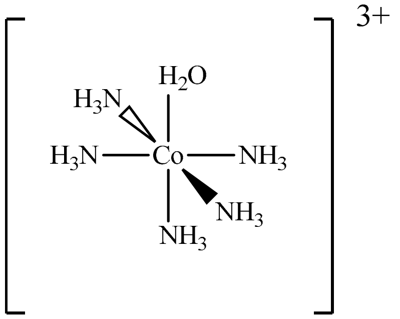

Imaginary frequencies: none
E^0: -1740.5644836 a.u.

Co	0.022006000	0.029319000	0.000000000
N	-0.012523000	-0.031046000	1.998911000
N	-1.167931000	1.630333000	0.000000000
O	-1.603162000	-1.151635000	0.000000000
N	-0.012523000	-0.031046000	-1.998911000
N	1.587523000	1.211854000	0.000000000
H	-0.106564000	-0.982702000	-2.362368000
H	-0.106564000	-0.982702000	2.362368000
H	1.793974000	-1.698327000	0.808207000
H	0.599921000	-2.443543000	0.000000000
H	-1.068893000	2.251174000	-0.806073000
H	-2.151063000	1.348354000	0.000000000
H	-1.068893000	2.251174000	0.806073000
H	1.628948000	1.838272000	-0.807146000
H	1.628948000	1.838272000	0.807146000
H	2.485427000	0.722323000	0.000000000
H	-2.094753000	-1.466716000	-0.773258000
H	-2.094753000	-1.466716000	0.773258000
H	1.793974000	-1.698327000	-0.808207000
H	-0.787083000	0.483682000	2.424397000
H	0.822611000	0.331210000	-2.464741000
H	-0.787083000	0.483682000	-2.424397000
H	0.822611000	0.331210000	2.464741000
N	1.175862000	-1.598135000	0.000000000

Complex **12**:

cis-$[Co(NH_3)_4(NO_2)_2]^+$

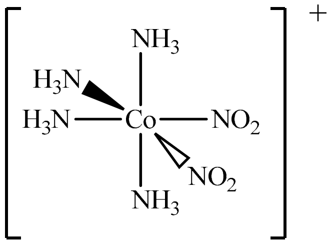

Imaginary frequencies: none
E^0: -2018.5149459 a.u.

Co	0.075118000	0.008575000	0.000000000
N	0.024230000	0.011628000	1.965435000
N	-1.032702000	1.606567000	0.000000000
N	-1.486150000	-1.151954000	0.000000000
N	0.024230000	0.011628000	-1.965435000
N	1.649744000	1.295603000	0.000000000
H	-0.641239000	-0.725288000	-2.218160000
H	-0.641239000	-0.725288000	2.218160000
H	1.806894000	-1.792079000	0.812743000
H	0.599764000	-2.506358000	0.000000000
O	-1.331840000	2.111131000	-1.064323000
O	-1.331840000	2.111131000	1.064323000
H	1.609565000	1.912408000	-0.808467000
H	1.609565000	1.912408000	0.808467000
H	2.578902000	0.883647000	0.000000000
O	-1.932399000	-1.532920000	-1.064139000
O	-1.932399000	-1.532920000	1.064139000
H	1.806894000	-1.792079000	-0.812743000
H	-0.427434000	0.898549000	2.211914000
H	0.874275000	-0.095478000	-2.509336000
H	-0.427434000	0.898549000	-2.211914000
H	0.874275000	-0.095478000	2.509336000
N	1.205570000	-1.687601000	0.000000000

Complex **13**:

cis-$[Co(NH_3)_4CO_3]^+$

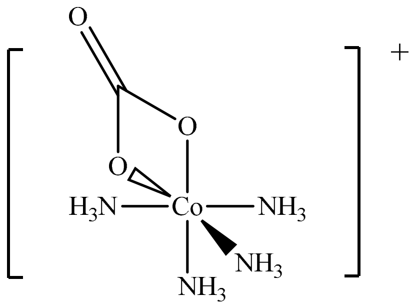

Imaginary frequencies: none
E^0: -1872.1601993 a.u.

Co	0.000000000	0.000000000	0.420919000
N	-1.970179000	0.000352000	0.286030000
N	0.000609000	1.553293000	1.716830000
C	0.000000000	0.000000000	-1.887349000
N	1.970179000	-0.000352000	0.286030000
N	-0.000609000	-1.553293000	1.716830000
H	2.209143000	0.812572000	-0.279575000
H	-2.209011000	0.814056000	-0.278508000
H	0.807219000	1.643129000	2.328049000
H	0.008068000	2.369952000	1.107028000
H	-0.812935000	1.650909000	2.317583000
H	0.812935000	-1.650909000	2.317583000
H	-0.807219000	-1.643129000	2.328049000
H	-0.008068000	-2.369952000	1.107028000
O	-0.000302000	-1.072229000	-1.076774000
O	0.000302000	1.072229000	-1.076774000
H	-2.569570000	-0.000197000	1.105853000
H	2.209011000	-0.814056000	-0.278508000
H	2.569570000	0.000197000	1.105853000
H	-2.209143000	-0.812572000	-0.279575000
O	0.000000000	0.000000000	-3.077294000

Complex **14**:

cis-$[Co(NH_3)_4(H_2O)Cl]^{2+}$

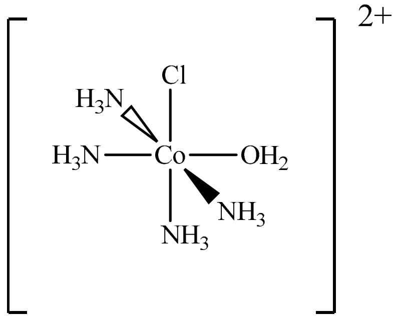

Imaginary frequencies: none
E^0: -2144.6381151 a.u.

Co	0.067261000	-0.078576000	0.000000000
N	0.010785000	-0.064503000	1.983634000
Cl	-1.313751000	1.617516000	0.000000000
O	-1.485795000	-1.335136000	0.000000000
N	0.010785000	-0.064503000	-1.983634000
N	1.523488000	1.220600000	0.000000000
H	-0.305384000	-0.932151000	-2.416430000
H	-0.305384000	-0.932151000	2.416430000
H	1.885626000	-1.788157000	0.809434000
H	0.691521000	-2.534978000	0.000000000
H	1.437767000	1.846372000	-0.800365000
H	1.437767000	1.846372000	0.800365000
H	2.477321000	0.860825000	0.000000000
H	-2.081511000	-1.238162000	-0.757516000
H	-2.081511000	-1.238162000	0.757516000
H	1.885626000	-1.788157000	-0.809434000
H	-0.655453000	0.662881000	2.252682000
H	0.885813000	0.161640000	-2.454460000
H	-0.655453000	0.662881000	-2.252682000
H	0.885813000	0.161640000	2.454460000
N	1.273496000	-1.696742000	0.000000000

Complex **15**:

cis-$[Co(NH_3)_4(H_2O)_2]^{3+}$

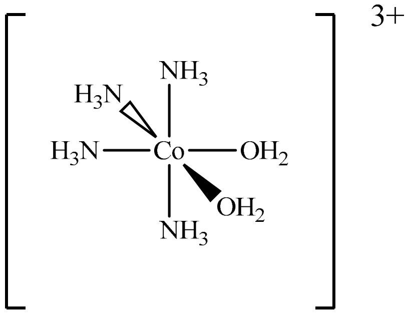

Imaginary frequencies: none
E^0: -1995.222318 a.u.

Co	0.076088000	0.011288000	0.000000000
N	0.051166000	-0.034197000	1.995455000
O	-1.046697000	1.659797000	0.000000000
O	-1.543015000	-1.151847000	0.000000000
N	0.051166000	-0.034197000	-1.995455000
N	1.634483000	1.192393000	0.000000000
H	-0.171368000	-0.960992000	-2.368901000
H	-0.171368000	-0.960992000	2.368901000
H	1.783277000	-1.717155000	0.810808000
H	0.560391000	-2.429628000	0.000000000
H	-1.451647000	2.085633000	-0.770226000
H	-1.451647000	2.085633000	0.770226000
H	1.636435000	1.822704000	-0.805402000
H	1.636435000	1.822704000	0.805402000
H	2.552040000	0.740572000	0.000000000
H	-2.119614000	-1.279651000	-0.769350000
H	-2.119614000	-1.279651000	0.769350000
H	1.783277000	-1.717155000	-0.810808000
H	-0.638750000	0.586471000	2.426117000
H	0.934520000	0.214889000	-2.447306000
H	-0.638750000	0.586471000	-2.426117000
H	0.934520000	0.214889000	2.447306000
N	1.169829000	-1.606464000	0.000000000

Complex **16**:

mer-[Co(NH_3)$_3$(NO_2)$_3$]

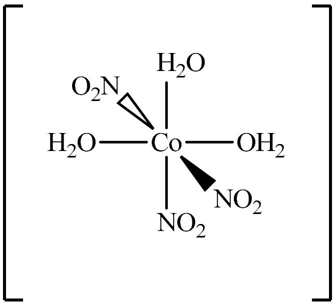

Imaginary frequencies: none
E^0: -2167.2171075 a.u.

Co	-0.020286000	0.041338000	0.000000000
N	-0.049532000	0.080705000	1.946196000
N	-1.184333000	1.609178000	0.000000000
N	-1.540853000	-1.200716000	0.000000000
N	-0.049532000	0.080705000	-1.946196000
N	1.626147000	1.117264000	0.000000000
H	-0.473646000	-0.755040000	-2.337206000
H	-0.473646000	-0.755040000	2.337206000
H	1.802092000	-1.559541000	0.813641000
H	0.680927000	-2.435781000	0.000000000
O	-1.541841000	2.090188000	-1.061236000
O	-1.541841000	2.090188000	1.061236000
O	2.161194000	1.390489000	-1.063640000
O	2.161194000	1.390489000	1.063640000
O	-1.964095000	-1.625901000	-1.063965000
O	-1.964095000	-1.625901000	1.063965000
H	1.802092000	-1.559541000	-0.813641000
H	-0.668038000	0.875489000	2.141885000
H	0.867382000	0.295232000	-2.328151000
H	-0.668038000	0.875489000	-2.141885000
H	0.867382000	0.295232000	2.328151000
N	1.194536000	-1.559353000	0.000000000

Complex **17**:

fac-[$Co(NH_3)_3(NO_2)_3$]

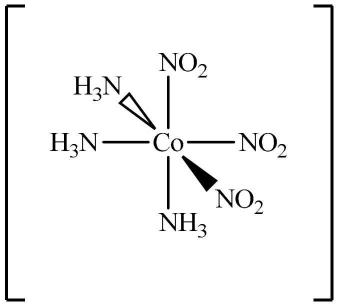

Imaginary frequencies: none
E^0: -2167.1986573 a.u.

Co	0.015886000	0.035364000	-0.018310000
N	0.145932000	0.066918000	1.906324000
N	-1.102134000	1.607844000	-0.093333000
N	-1.519365000	-1.135637000	-0.020459000
N	-0.027052000	-0.012052000	-2.016329000
N	1.552936000	1.330558000	-0.031383000
H	-0.658737000	-0.773718000	-2.264611000
O	0.077414000	-0.985438000	2.518114000
H	0.980615000	-1.971473000	1.014492000
H	0.621211000	-2.326486000	-0.551426000
O	-0.849282000	2.395932000	-1.019509000
O	-1.963887000	1.818134000	0.708777000
H	1.292689000	2.079668000	-0.670133000
H	1.540007000	1.716836000	0.915792000
H	2.492945000	1.017994000	-0.244554000
O	-1.517264000	-2.012879000	-0.899864000
O	-2.400479000	-1.035647000	0.781821000
H	2.087449000	-1.649803000	-0.174850000
O	0.399323000	1.121816000	2.461992000
H	0.855701000	-0.156159000	-2.492353000
H	-0.410820000	0.886959000	-2.308544000
N	1.099103000	-1.656568000	0.048433000

Complex **18**:

mer-$[Co(NH_3)_3(H_2O)_3]^{3+}$

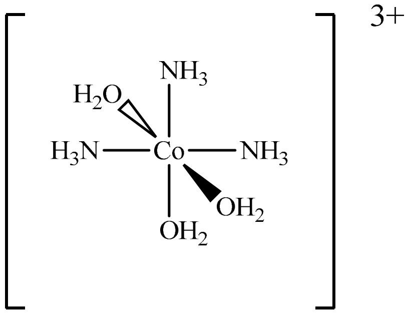

Imaginary frequencies: none
E^0: -1780.2290381 a.u.

Co	0.017241000	0.011583000	0.000000000
N	-0.004953000	-0.026554000	1.992105000
O	-1.164128000	1.609625000	0.000000000
O	-1.533073000	-1.164161000	0.000000000
N	-0.004953000	-0.026554000	-1.992105000
O	1.613643000	1.120513000	0.000000000
H	-0.144546000	-0.968193000	-2.370015000
H	-0.144546000	-0.968193000	2.370015000
H	1.799357000	-1.594192000	0.807887000
H	0.637468000	-2.425648000	0.000000000
H	-1.403419000	2.146504000	-0.770611000
H	-1.403419000	2.146504000	0.770611000
H	1.905019000	1.632719000	-0.771205000
H	1.905019000	1.632719000	0.771205000
H	-2.118022000	-1.246523000	-0.771004000
H	-2.118022000	-1.246523000	0.771004000
H	1.799357000	-1.594192000	-0.807887000
H	-0.748513000	0.534918000	2.415992000
H	0.854235000	0.299753000	-2.442820000
H	-0.748513000	0.534918000	-2.415992000
H	0.854235000	0.299753000	2.442820000
N	1.173397000	-1.553155000	0.000000000

Complex **19**:

fac-$[Co(NH_3)_3(H_2O)_3]^{3+}$

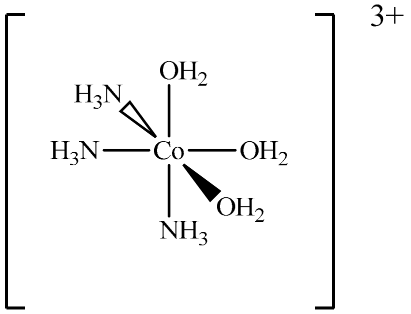

Imaginary frequencies: none
E^0: -1780.2421265 a.u.

Co	0.059207000	0.028792000	-0.063258000
O	0.013992000	0.033143000	1.916278000
O	-1.097437000	1.633260000	0.029982000
O	-1.612067000	-1.029348000	0.023152000
N	-0.025123000	0.049542000	-2.007342000
N	1.652612000	1.145980000	-0.054219000
H	0.706611000	-0.479521000	-2.488486000
H	-0.675235000	-0.441807000	2.406830000
H	1.197740000	-1.967741000	0.892560000
H	0.722611000	-2.360803000	-0.620503000
H	-1.476262000	2.189454000	-0.668372000
H	-1.350314000	2.024347000	0.881072000
H	2.194167000	1.152316000	-0.922275000
H	1.393300000	2.122535000	0.112752000
H	2.326469000	0.906338000	0.678183000
H	-1.762570000	-1.987386000	0.015263000
H	-2.481513000	-0.599385000	0.044010000
H	2.084082000	-1.504070000	-0.393237000
H	0.618696000	0.420506000	2.568081000
H	0.011465000	0.987106000	-2.416696000
H	-0.909600000	-0.359128000	-2.322749000
N	1.121362000	-1.601964000	-0.060937000

Complex **20**:

cis-$[Co(NH_3)_2(NO_2)_4]^-$

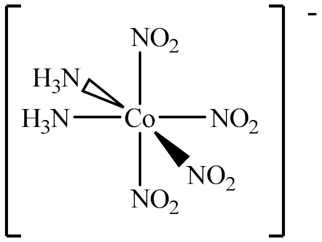

Imaginary frequencies: none
E^0: -2315.7674034 a.u.

Co	0.013169000	0.095560000	0.032053000
N	0.264777000	0.056632000	1.976300000
N	-1.244123000	1.600096000	0.011904000
N	-1.479288000	-1.174894000	0.094134000
N	-0.099565000	0.096105000	-1.932930000
N	1.576541000	1.262414000	-0.165570000
H	-0.631731000	-0.720223000	-2.221873000
H	1.284149000	-1.751392000	0.924221000
H	0.766831000	-2.208931000	-0.603845000
O	-1.336685000	2.251764000	-1.041452000
O	-1.938107000	1.853808000	0.958785000
O	2.487447000	0.838162000	-0.899667000
O	1.656450000	2.345696000	0.356038000
O	-1.525733000	-2.010645000	-0.826940000
O	-2.294671000	-1.186166000	0.981148000
H	2.089597000	-1.175721000	-0.452954000
O	0.109641000	1.037907000	2.651264000
H	0.829968000	0.110584000	-2.337679000
H	-0.593213000	0.973162000	-2.132530000
O	0.645400000	-1.012543000	2.480739000
N	1.200435000	-1.471609000	-0.059411000

Complex **21**:

cis-$[Co(NH_3)_2(CO_3)_2]^-$

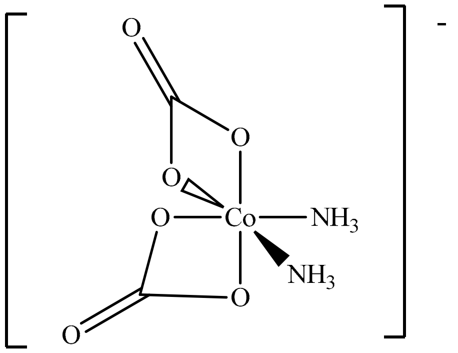

Imaginary frequencies: none
E^0: -2023.0443409 a.u.

Co	-1.509417000	1.720395000	0.267683000
N	-1.143975000	1.549230000	2.220895000
C	-0.120716000	-0.112193000	-0.029825000
N	-1.140658000	3.673830000	0.106208000
H	-1.271512000	0.541054000	2.289226000
H	-1.387798000	3.771977000	-0.876971000
H	-1.742580000	4.274689000	0.655474000
H	-0.167006000	3.919469000	0.235090000
O	0.252298000	1.155596000	-0.106177000
O	-1.414821000	-0.158225000	0.252026000
H	-1.824443000	2.015526000	2.807820000
H	-0.200125000	1.778785000	2.506351000
O	0.610908000	-1.077014000	-0.158073000
O	-3.352322000	2.088099000	0.443404000
O	-2.305139000	1.897209000	-1.427611000
C	-3.487449000	2.122527000	-0.872964000
O	-4.518507000	2.360522000	-1.475356000

Complex **22**:

cis,mer-[Co(NH_3)$_2$(NO_2)$_3$$CH_3$]$^-$

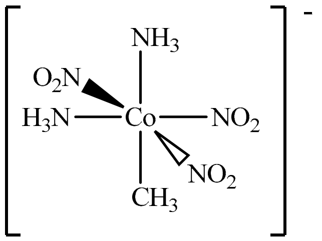

Imaginary frequencies: none
E^0: -2150.6025633 a.u.

Co	-1.111205000	0.753679000	0.000000000
N	-1.282655000	0.830571000	1.969458000
N	-3.090832000	1.056437000	0.000000000
C	-1.401837000	-1.178629000	0.000000000
N	-1.282655000	0.830571000	-1.969458000
N	-0.773531000	2.780844000	0.000000000
O	-0.959839000	-0.036778000	-2.742945000
O	-0.959839000	-0.036778000	2.742945000
O	1.399593000	0.559486000	1.063957000
H	-3.312463000	1.583394000	-0.840991000
H	-3.601202000	0.181816000	0.000000000
H	-3.312463000	1.583394000	0.840991000
H	-1.208214000	3.134994000	-0.847644000
H	-1.208214000	3.134994000	0.847644000
H	0.210926000	3.017175000	0.000000000
H	-1.952210000	-1.467108000	-0.900460000
H	-1.952210000	-1.467108000	0.900460000
O	1.399593000	0.559486000	-1.063957000
O	-1.867164000	1.852975000	-2.399708000
O	-1.867164000	1.852975000	2.399708000
N	0.795922000	0.558623000	0.000000000
H	-0.446115000	-1.705740000	0.000000000

Complex **23**:

cis,fac-$[Co(NO_2)_3(NH_3)_2CH_3]^-$

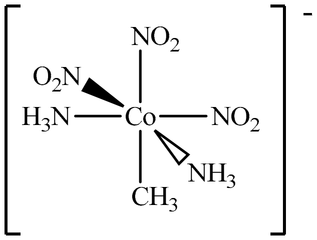

Imaginary frequencies: none
E^0: -2150.6041769 a.u.

Co	-1.187328000	0.693569000	-0.067428000
N	-1.162575000	0.606210000	1.875590000
N	-3.217621000	0.898548000	-0.099084000
N	-1.498002000	-1.255034000	-0.058687000
N	-1.338576000	0.687740000	-2.043891000
N	-0.820401000	2.609044000	-0.203850000
H	-0.573356000	0.266795000	-2.553727000
O	-0.974314000	-0.505979000	2.411190000
H	1.245438000	1.093713000	0.589677000
H	1.001641000	-0.623026000	0.212086000
O	-3.832052000	1.752871000	0.495754000
O	-3.839424000	0.074656000	-0.811578000
O	-1.217173000	3.190631000	-1.232201000
O	-0.180066000	3.218012000	0.618326000
H	-0.867507000	-1.788261000	-0.641703000
H	-2.466393000	-1.407534000	-0.335253000
H	1.173543000	0.571295000	-1.102277000
O	-1.364916000	1.576334000	2.559602000
H	-1.382222000	1.701716000	-2.220260000
H	-2.226454000	0.253936000	-2.283499000
C	0.759846000	0.399991000	-0.099196000
H	-1.333703000	-1.469690000	0.934102000

Complex **24**:

trans,*mer*-[Co$(NH_3)_2(NO_2)_3CH_3$]$^-$

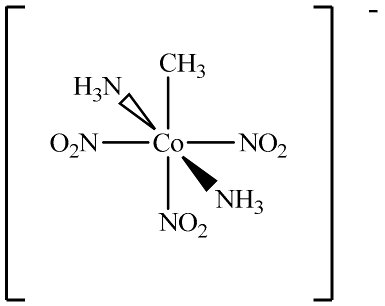

Imaginary frequencies: none
E^0: -2150.6363178 a.u.

Co	-0.001198000	0.014630000	0.000000000
N	-0.040610000	0.066517000	1.936042000
N	-1.217908000	1.660873000	0.000000000
N	-1.527303000	-1.207721000	0.000000000
N	-0.040610000	0.066517000	-1.936042000
N	1.628960000	1.096288000	0.000000000
H	-0.459392000	-0.784873000	-2.292840000
H	-0.459392000	-0.784873000	2.292840000
H	1.838767000	-1.538460000	0.883976000
H	0.626103000	-2.492564000	0.000000000
O	-1.588229000	2.161166000	-1.060401000
O	-1.588229000	2.161166000	1.060401000
O	2.158331000	1.417428000	-1.060871000
O	2.158331000	1.417428000	1.060871000
O	-1.992929000	-1.615614000	-1.060697000
O	-1.992929000	-1.615614000	1.060697000
H	1.838767000	-1.538460000	-0.883976000
H	-0.648848000	0.863705000	2.142796000
H	0.893695000	0.250851000	-2.284778000
H	-0.648848000	0.863705000	-2.142796000
H	0.893695000	0.250851000	2.284778000
C	1.192821000	-1.557616000	0.000000000

Complex **25**:

$[Co(NH_3)_6]^{3+}$

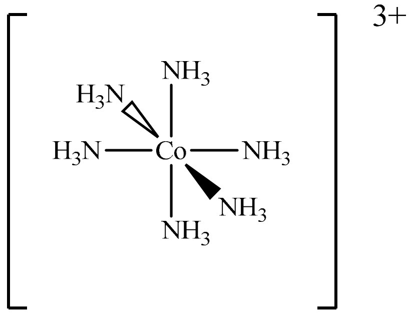

Imaginary frequencies: none
E^0: -1720.7248341 a.u.

Co	-1.242128000	0.756437000	0.000000000
N	-1.211505000	0.715995000	2.004476000
N	-3.220107000	1.088384000	0.000000000
N	-1.581582000	-1.221094000	0.000000000
N	-1.211505000	0.715995000	-2.004476000
N	-0.980601000	2.744367000	0.000000000
H	-0.647906000	-0.050250000	-2.379566000
H	-0.647906000	-0.050250000	2.379566000
H	1.216882000	0.917434000	0.807690000
H	1.068691000	-0.469256000	0.000000000
H	-3.551561000	1.623842000	-0.805203000
H	-3.799650000	0.246344000	0.000000000
H	-3.551561000	1.623842000	0.805203000
H	-1.396347000	3.217345000	-0.805185000
H	-1.396347000	3.217345000	0.805185000
H	-0.006155000	3.053286000	0.000000000
H	-2.116858000	-1.547428000	-0.807607000
H	-2.116858000	-1.547428000	0.807607000
H	1.216882000	0.917434000	-0.807690000
H	-2.125140000	0.595966000	2.447084000
H	-0.823972000	1.552610000	-2.445919000
H	-2.125140000	0.595966000	-2.447084000
H	-0.823972000	1.552610000	2.445919000
N	0.748243000	0.501194000	0.000000000
H	-0.747394000	-1.811355000	0.000000000

Complex **26**:

trans-$[Co(NH_3)_4(NO_2)_2]^+$

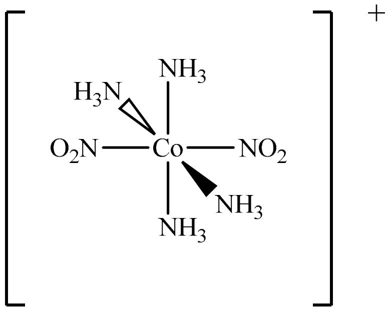

Imaginary frequencies: none
E^0: -2018.5139612 a.u.

Co	-0.037849000	0.030218000	0.000000000
N	-0.074982000	-0.003727000	1.965397000
N	-1.308345000	1.548898000	0.000000000
N	-1.548859000	-1.267310000	0.000000000
N	-0.074982000	-0.003727000	-1.965397000
N	1.435787000	1.286939000	0.000000000
H	-0.812851000	-0.667288000	-2.225308000
H	-0.812851000	-0.667288000	2.225308000
H	1.882378000	-1.444071000	0.806684000
H	0.809432000	-2.374699000	0.000000000
H	-1.191340000	2.157872000	-0.806372000
H	-2.280085000	1.245002000	0.000000000
H	-1.191340000	2.157872000	0.806372000
O	1.876341000	1.661872000	-1.069907000
O	1.876341000	1.661872000	1.069907000
O	-2.000426000	-1.657945000	-1.064791000
O	-2.000426000	-1.657945000	1.064791000
H	1.882378000	-1.444071000	-0.806684000
H	-0.267645000	0.898557000	2.389272000
H	0.806294000	-0.282650000	-2.385568000
H	-0.267645000	0.898557000	-2.389272000
H	0.806294000	-0.282650000	2.385568000
N	1.262974000	-1.463138000	0.000000000

Complex **27**:

trans-$[Co(NH_3)_2(NO_2)_4]^-$

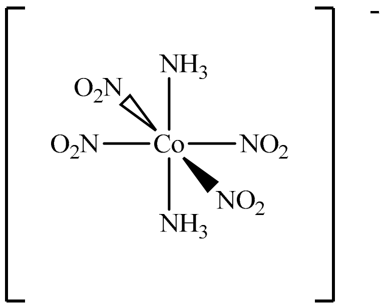

Imaginary frequencies: none
E^0: -2315.7950751 a.u.

Co	-1.304667000	0.841291000	0.000000000
N	-1.322889000	0.865513000	1.935272000
N	-3.274129000	1.110182000	0.000000000
N	-1.577018000	-1.095081000	0.000000000
N	-1.322889000	0.865513000	-1.935272000
N	-0.984831000	2.802828000	0.000000000
H	-0.763541000	0.104076000	-2.296459000
H	-0.763541000	0.104076000	2.296459000
O	1.218596000	0.437584000	1.064381000
O	-3.879802000	1.187210000	-1.060759000
O	-3.879802000	1.187210000	1.060759000
O	-0.880979000	3.404403000	-1.060788000
O	-0.880979000	3.404403000	1.060788000
O	-1.656976000	-1.690013000	-1.064354000
O	-1.656976000	-1.690013000	1.064354000
O	1.218596000	0.437584000	-1.064381000
H	-2.297294000	0.783342000	2.216900000
H	-0.954279000	1.770839000	-2.217879000
H	-2.297294000	0.783342000	-2.216900000
H	-0.954279000	1.770839000	2.217879000
N	0.626389000	0.535130000	0.000000000
